# High‐Asymmetry Metasurface: A New Solution for Terahertz Resonance via Active Learning‐Augmented Diffusion Model

**DOI:** 10.1002/advs.202508610

**Published:** 2025-09-23

**Authors:** Qiqi Dai, Yinpeng Wang, Cheng Xu, Dongxiao Li, Prakash Pitchappa, Thomas Caiwei Tan, Ranjan Singh, Chengkuo Lee

**Affiliations:** ^1^ Department of Electrical & Computer Engineering National University of Singapore 4 Engineering Drive 3 Singapore 117576 Republic of Singapore; ^2^ Center for Intelligent Sensors and MEMS (CISM) National University of Singapore 5 Engineering Drive 1 Singapore 117608 Republic of Singapore; ^3^ Institute of Microelectronics (IME), Agency for Science Technology and Research (A*STAR) 2 Fusionopolis Way, Innovis #08‐02 Singapore 138634 Republic of Singapore; ^4^ Division of Physics and Applied Physics, School of Physical and Mathematical Sciences Nanyang Technological University Singapore 637371 Republic of Singapore; ^5^ Department of Electrical Engineering University of Notre Dame Notre Dame IN 46556 USA; ^6^ National Centre for Advanced Integrated Photonics (NCAIP) Singapore 639798 Republic of Singapore

**Keywords:** diffusion model, high‐asymmetry structures, high‐FoM resonance, inverse design, physics‐constrained active learning, terahertz metamaterials

## Abstract

Terahertz (THz) metamaterials with high‐figure‐of‐merit (high‐FoM) performance resonance are essential for advancing sensors, detectors, and imagers. Conventional designs focus on symmetric or low‐asymmetry geometric structures, leaving high‐asymmetry designs largely unexplored due to the inefficiency of trial‐and‐error‐based rational design. Recent deep learning techniques offer automation and acceleration but are constrained by the need for large datasets inherent to their data‐driven nature. Here, a novel prior knowledge‐guided generative model augmented by a physics‐constrained active learning mechanism to design high‐asymmetry metamaterials. An advanced diffusion model learns features from a small set of classical structures with high‐FoM THz resonance and generates new high‐asymmetry structures. To mitigate the limited number of classical structures, the generated high‐asymmetry structures are actively selected and integrated into the initial training dataset based on their physical characteristics. Experimental results demonstrate the superior resonance performance of the generated high‐asymmetry metamaterials over classical designs, exhibiting improvements exceeding 30% in key resonance metrics. Remarkably, this performance is attained using only 68 classical structures as the initial training dataset, significantly reducing the data requirements for deep learning‐based metamaterial design. The proposed scheme for generating high‐asymmetry structures provides a new effective and efficient solution for high‐FoM resonance, expanding applications in high‐sensitivity THz metadevices.

## Graphical abstract

A prior knowledge‐guided diffusion model augmented by physics‐constrained active learning is developed to design high‐asymmetry terahertz metamaterials. Trained on only a small set of classical structures, the model efficiently generates new high‐metrics designs. Experimental results confirm notable improvements and reveal multi‐resonance phenomena, highlighting the method's capability to overcome data limitations and discover novel metamaterial solutions.

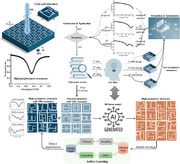

## 1 Introduction

Terahertz (THz) electromagnetic waves (0.1–10 THz) occupy the transitional spectral region between microwave and infrared radiation, thereby inheriting advantageous characteristics from both neighboring bands.^[^ ^1^, ^2^ ^]^ The engineering of THz metamaterials is fundamental to the efficient control and manipulation of THz wave propagation, enabling a wide range of advanced applications such as the next generation of wireless communications, sensing, high‐resolution sensing, quantum information processing, imaging, and spectroscopy.^[^ ^3^, ^4^, ^5^, ^6^ ^]^ The attainment of high‐performance resonances is particularly crucial for enhancing the metamaterials’ sensitivity and functionality.^[^ ^7^, ^8^, ^9^, ^10^ ^]^ Among the earliest and most extensively investigated metamaterial configurations for THz resonance were split‐ring resonators (SRRs) based on an inductive‐capacitive (LC) resonance mechanism.^[^ ^11^, ^12^, ^13^ ^]^ Nevertheless, critical THz resonance characteristics, such as the quality factor (Q), spectral sharpness, and field enhancement, still required significant improvement to meet the stringent demands of emerging high‐performance THz metadevices.

Conventional studies involving rational design have primarily focused on designing THz metamaterial structures with symmetric or low‐asymmetry geometries to support well‐defined resonant modes with augmented resonance performance.^[^ ^14^, ^15^, ^16^, ^17^, ^18^, ^19^, ^20^, ^21^, ^22^, ^23^, ^24^, ^25^, ^26^, ^27^, ^28^, ^29^, ^30^, ^31^, ^32^, ^33^, ^34^, ^35^, ^36^ ^]^ These physics‐driven design methods rely on well‐established physical mechanisms, such as Fano resonance, bound states in the continuum (BIC), electromagnetically induced transparency (EIT), and toroidal dipoles. To excite the Fano resonance, two individual asymmetric D‐split resonators (ADSRs) were combined as a unit cell to suppress radiation losses and improve the Q factor.^[^ ^15^, ^16^ ^]^ Similarly, SRRs with dual capacitive gaps at asymmetric positions, enabling two unequal metallic wires to form an asymmetric resonator, were developed to produce Fano resonance with a figure of merit (FoM) that considers the trade‐off between the Q factor and resonance intensity.^[^ ^24^, ^25^, ^26^ ^]^ Furthermore, the coupling coefficient between two opposite nested metallic SRRs was optimized to satisfy the Friedrich‐Wintgen BIC.^[^ ^15^, ^16^, ^17^ ^]^ Another type of symmetry‐protected BIC arose by the side coupled configuration with the resonating arms flipped.^[^ ^21^ ^]^ To realize the EIT effect, two sub‐resonators, a fork‐shaped resonator (FSR) and a U‐shaped SRR, were merged to form the two different “big‐bright” resonance modes.^[^ ^28^ ^]^ By symmetrically moving the pair of capacitive gaps toward central branch of SRRs, sharp toroidal dipolar response was demonstrated to enhance the THz resonance performance.^[^ ^31^ ^]^ More introductions for THz metamaterials can be found in Note S1 (Supporting Information). However, all these works involve symmetric structures or slightly breaking the symmetry of the pattern via tuning one or two asymmetry factor of the unit cell to optimize the THz resonance performance. When the asymmetry of the geometric pattern increases and additional asymmetry factors are incorporated, the degree of freedom (DoF) of the structure expands significantly, resulting in highly increased complexity in the rational design process.

In recent years, to address the limitations of rational design, deep learning techniques have been developed for the inverse design of metamaterial structures due to the powerful learning capability and computational superiority of artificial intelligence.^[^ ^37^, ^38^, ^39^, ^40^ ^]^ Different from the rational design methods driven by specific physical mechanisms, the data‐driven design methods use deep learning to extract features from provided dataset and automatically design new structures through trained models. These models do not incorporate physical principles explicitly but instead learn from the statistical distribution of the training set. As such, the quality and diversity of the generated designs are strongly dependent on the richness of the input data. Starting from optimizing geometric parameters of a specific structure, 1D neural networks have been applied to find the mapping relationship between the geometric parameters and the optical response, and then the geometric parameters corresponding to the desired response can be estimated using the trained neural networks.^[^ ^41^, ^42^, ^43^, ^44^, ^45^, ^46^ ^]^ However, the geometric parameter optimization using 1D neural networks was usually based on one fixed geometry pattern, limiting the design space of metamaterial structures to a single configuration (See Note S1, Supporting Information). To expand the DoF of metamaterial structure design, several 2D deep neural networks have been used to generate the structural image of geometry pattern corresponding to the desired response. First model, 2D convolutional neural networks (CNNs) were employed to describe the mapping relationship between the 2D structures and their phase or amplitude responses.^[^ ^47^, ^48^, ^49^ ^]^ Second model, generative adversarial networks (GANs), including a generator sub‐network and a discriminator sub‐network, utilized the adversarial learning between them to constrain the generator to generate structural images distributing close to those in the provided metamaterial dataset.^[^ ^50^, ^51^, ^52^, ^53^ ^]^ Third model, variational auto‐encoders (VAEs) connected the encoder network to the symmetric decoder through a probabilistic latent space and finally reconstructed the image space of metamaterial structures.^[^ ^53^, ^54^, ^55^ ^]^ Nevertheless, all these deep learning models were driven by large datasets and required a diverse set of labelled metamaterial structures to train the neural networks. For example, over ten thousand of antenna structures with their labels of the reflection spectrums were collected to train the GAN.^[^ ^51^ ^]^ The preparation process of a large training dataset for fitting the generative model is highly time‐consuming, which causes high computational costs in existing deep learning‐based inverse design. Besides, when the geometric shapes used in the training dataset are regular and limited, the generated structures via the trained network would be close to those in the training dataset, making it challenging to produce new high‐asymmetry metamaterial structures.

To solve the issues of existing deep learning‐based metamaterial design and fill the gap of high‐asymmetry metamaterial structure design, we propose a novel prior knowledge‐guided generative model to automatically generate high‐asymmetry metamaterials with THz resonance. Inspired by the rational design process that starts from literature review, the proposed generative model is trained to learn the features of classical THz metamaterial structures as the prior knowledge and then produce new high‐asymmetry structures. As existing structures can only form a small training dataset that limits the learning capability of the generative model, we introduce a physics‐constrained active learning mechanism to iteratively augment the training dataset using the newly generated structures, including 1) data query for screening the structures that potentially have THz resonance and are valuable to be annotated and 2) annotating the structures with high‐FoM THz resonance into the training data for the next iteration of learning loop. An advanced diffusion model is employed as the generative model to sample new high‐asymmetry structures. Both the numerical and experimental demonstrations of the generated high‐asymmetry metamaterial structures show the superior performance on THz resonance compared to traditional structures. The proposed scheme provides an efficient metamaterial structure design method based on deep learning with limited data and proves the high‐asymmetry metamaterial structures to be a powerful solution of THz resonance.

## 2 Results and Discussion

### 2.1 Design Mechanism with Physics‐Constrained Active Learning

#### 2.1.1 Proposed Design Mechanism for High‐Asymmetry Metamaterials

**Figure 1:**
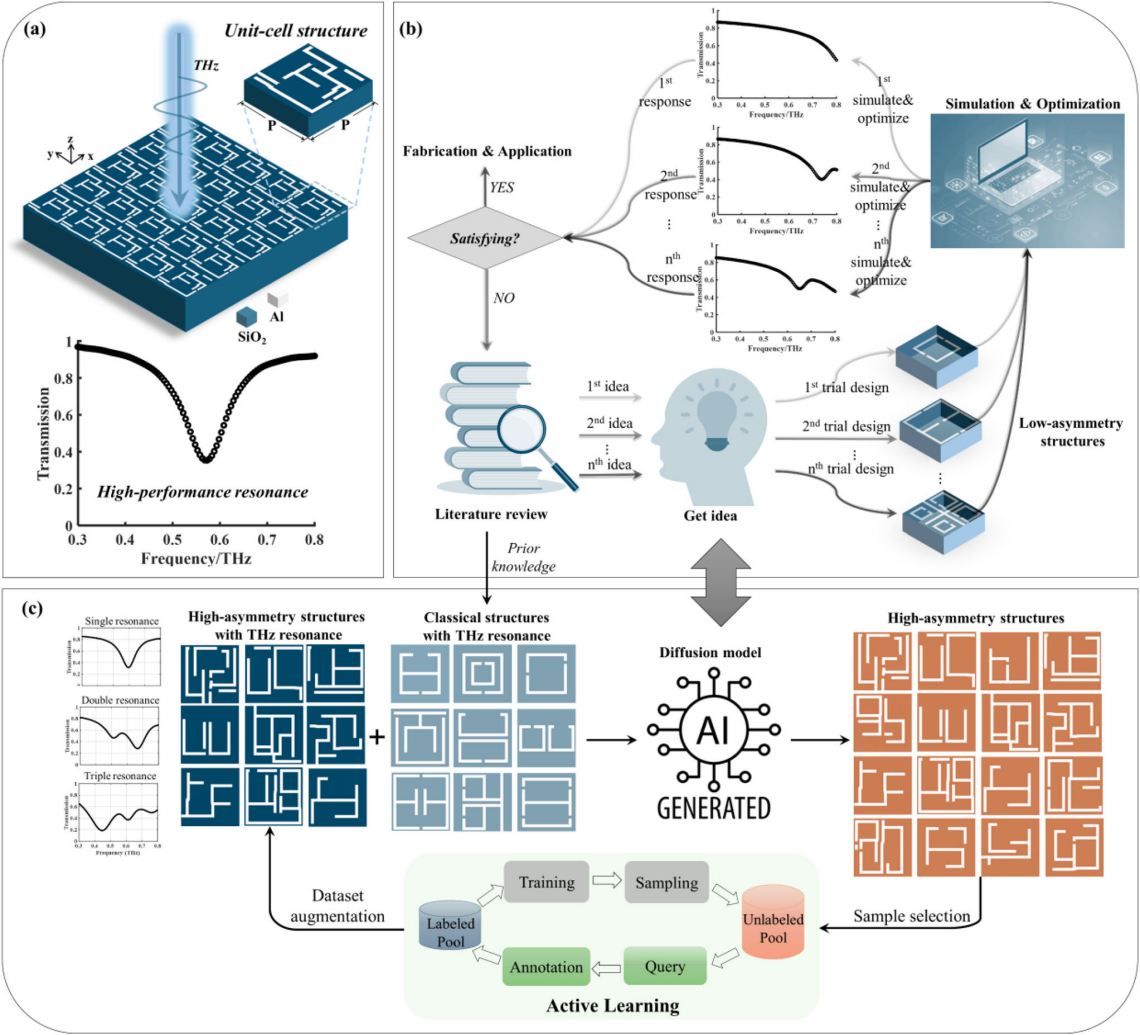
Schematic illustration of the proposed high‐asymmetry THz metamaterial structure design based on active learning‐augmented diffusion model. a) The target of realizing high‐FoM THz resonance via the metamaterial unit cell structure design. b) Rational design process of metamaterial structures via iterative trial‐and‐error optimization, which is limited in simple low‐asymmetry structure design. c) The proposed framework of generative model‐assisted high‐asymmetry structure design that is guided by prior knowledge obtained from literature review. The physics‐constrained active learning solves the issue of limited training data.

**Figure** **1** depicts the overview of the proposed design flow for high‐asymmetry metamaterial structures with THz resonance. As shown in Figure 1a, the design target is to realize high‐FoM THz resonance via the metamaterial with periodic unit‐cell structure design. The top metal layer in each unit‐cell structure is discretized into a 2D geometry pattern with 100 × 100 pixels and each pixel dimension is 1 × 1 µm^2^. Traditional rational design process for the geometric patterns of metamaterial structures usually starts with literature review, as shown in Figure 1b. Typically, human designers draw inspiration for new structural designs from existing studies. The performance of the proposed new structure is then checked via simulations. If the response of the new structure does not satisfy the desired specification, human designers optimize its geometric parameters or re‐design the structure until obtaining the desired response. On the one hand, this process is highly labor‐ and time‐consuming due to the repetitive optimization and trial‐and‐error design for various structures. On the other hand, as the human imagination is limited, it is difficult to design high‐asymmetry structures with complicated and irregular geometries. Although several deep learning‐based design methods have been investigated to automatically and rapidly generate structures with the desired properties, the requirements on the sufficiency and diversity of the training dataset are very high. Therefore, designing high‐asymmetry structures is challenging using traditional rational design methods and existing deep learning‐based design techniques.

As shown in Figure 1c, we propose an active learning‐augmented generative model that simulates the rational design process for high‐asymmetry structure design. Beginning with literature review, a small set of classical structures are collected as the initial training dataset to fit the generative model. As the high‐performance THz resonances of these classical structures have already been demonstrated in existing studies, they work as prior knowledge to guide the generative model to learn their underlying features and then generate new structures with THz resonances. However, the limited amount of existing structures results in weak generative capability due to underfitting issues in the training process. To augment the training dataset, we introduce a physics‐constrained active learning mechanism to fuse the generated structures into each training loop. Via the physics‐constrained data query and annotation, selected structures generated by the diffusion model are added into the training dataset and then high‐asymmetry structures with high‐FoM resonances are produced in the sampling process. **Table** **1** presents a comparative analysis for various existing 2D metamaterial structure designs assisted by deep learning. It can be observed that our scheme offers an effective and efficient learning strategy involving the smallest number of data samples for training the advanced diffusion model to design high‐DoF metamaterial structures. The proposed strategy enhances the THz resonance performance as demonstrated by comparative study and real experiments, providing an innovative approach for metamaterial structure design.

**Table 1:** Comparison of 2D metamaterial structure design assisted by deep learning.

Type	Frequency	DoF	Network	Training strategy	Initial samples	Total samples	Asym. ^c^ ^)^	Comp. ^d^ ^)^	Expe. ^e^ ^)^
Reflect^[^ ^47^ ^]^	9–11 GHz	2^8×8^	CNN ^a^ ^)^	From scratch ^b^ ^)^	–	70,000	No	No	Yes
Reflect^[^ ^48^ ^]^	10 GHz	2^8×8^	CNN	Transfer learning	–	20,000	No	No	Yes
Transmit^[^ ^50^ ^]^	170−600 THz	2^64×64^	GAN	From scratch	–	6,500	Yes	Yes	No
Reflect^[^ ^51^ ^]^	250–500 THz	2^64×64^	GAN	From scratch	–	10,150	Yes	Yes	No
Transmit^[^ ^52^ ^]^	5–10 µm	2^64×64^	GAN	From scratch	–	29,000	No	No	No
Reflect^[^ ^54^ ^]^	40–100 THz	2^64×64^	VAE	From scratch	–	10,000	Yes	Yes	No
Transmit^[^ ^55^ ^]^	3–5 µm	2^100×100^	VAE	From scratch	–	20,000	Yes	No	No
Reflect^[^ ^56^ ^]^	1–1.5 THz	2^30×30^	VAE	Cyclic iteration	3,000	51,000	Yes	Yes	Yes
Transmit ^Ours^	0.3–0.8 THz	2^100×100^ ↑	Diffusion model	Active learning	68 ↓	454 ↓	Yes	Yes	Yes

^a)^ “CNN” in this table represents 2D convolutional neural networks developed for image classification/regression tasks;

^b)^ “From scratch” means that the generative network is trained from scratch without any additional learning strategy to overcome the limited dataset;

^c)^ “Asym.” represents whether asymmetric unit‐cell structures are designed;

^d)^ “Comp.” means whether the comparative study between the generated structures and the conventional ones in the initial training dataset is provided;

^e)^ “Expe.” represents whether real device fabrication and experiments are conducted.

#### 2.1.2 Framework of Physics‐Constrained Active Learning

**Figure 2:**
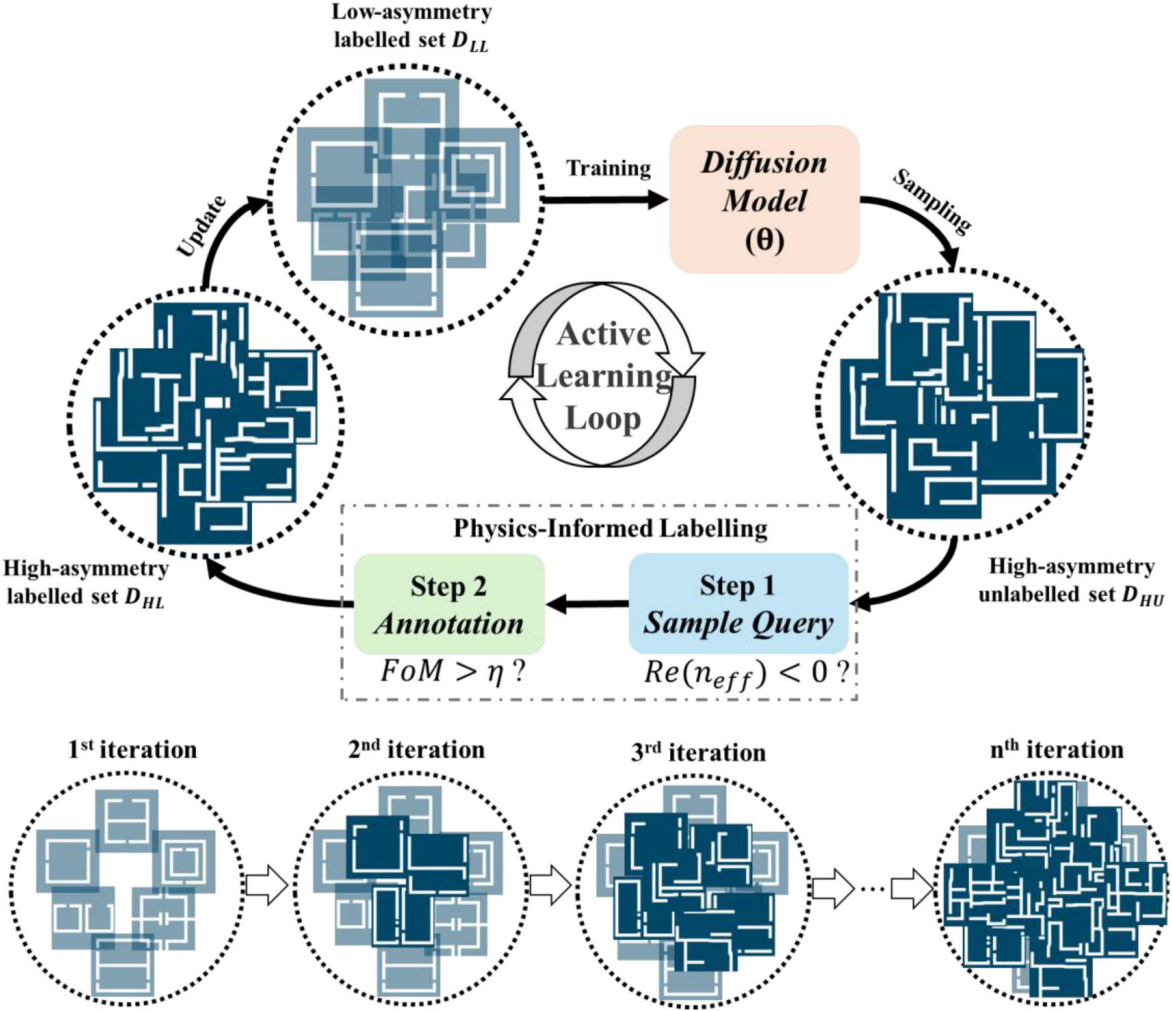
The proposed physics‐constrained active learning mechanism. A small set of existing low‐asymmetry structures *D_LL_* are collected from literature review as prior knowledge to initially train the generative diffusion model. Then in each loop of active learning, the generated high‐asymmetry structures with high‐FoM THz resonance *D_HL_* are selected and added into dataset for the following round of training. The selection mechanism involves a physics‐constrained labelling process that includes a sample query step according to the simulated effective refractive index and an annotation step according to the calculated FoM. After *n* iterations of active learning, the generated structures are in high asymmetry with high‐FoM THz resonance.

An active learning loop usually consists of two main steps: the first step is the data query to select samples that are valuable to be annotated, and the second step is the annotation to screen out the samples with the desired properties.^[^ ^57^, ^58^ ^]^ Instead of general scoring strategies used in data query, we propose a physics‐constrained active learning framework that enables the generative model to learn from limited datasets with THz resonance. The framework of the proposed active learning mechanism is shown in **Figure** **2**. We first collect a small set of labelled classical metamaterial structures to form the initial training dataset *D_LL_* and fit them into the generative model, which involves an advanced diffusion model with trainable parameters θ. In the active learning process, *D_LL_* guides the diffusion model to generate a high‐asymmetry unlabeled structure set *D_HU_*. Here, “labeled” and “unlabeled” mean that the structure has been verified with THz resonance or not. Initially, the asymmetry of the generated structures is still limited due to the small training dataset fully containing low‐asymmetry structures. To efficiently empower the training dataset, we design a physics‐constrained labelling strategy to select high‐asymmetry structures generated by the diffusion model, which includes two steps, 1) the data query based on effective refractive index *n_eff_* and 2) the annotation based on FoM of the resonance. In the first step, the high‐asymmetry structures with potential resonances are distinguished according to *n_eff_*, which is obtained via^[^ ^59^ ^]^

(1) $$n_{eff}=\frac{1}{kd}\;\cos^{-1}\left(\frac{1-S_{11}^{2}+S_{21}^{2}}{2S_{21}}\right)$$

*k*, *d*, *S*_11_, and *S*_21_ represent the wavenumber, metamaterial span, reflection, and transmission coefficients of the metamaterial, respectively. When a negative real part of *n_eff_* (*Re*(*n_eff_*) < 0) appears in the working frequency range of 0.3–0.8 THz, we screen the structure out as the one with potential THz resonance.^[^ ^60^ ^]^ This physics‐constrained query step reduces the workload of data annotation in the next step. In the second step, we calculate the FoM of the structure selected in the first step to evaluate its resonance performance, which is expressed as *FoM*  =  *Q* × *I*, where *Q*  = *f_r_*/*FWHM* , *f_r_* is the resonant frequency, *FWHM* measures the full width half maximum of the resonant peak, and *I* is the contrast intensity between the maximum and minimum value of each resonant peak.^[^ ^20^, ^21^, ^22^ ^]^ The generated structures with metrics larger than the defined threshold η are finally selected into the labelled high‐asymmetry structure set *D_HL_*, which is then combined with *D_LL_* as the new training dataset in the next iteration of active learning loop (See Note S2 and Figure S1, Supporting Information). As the iteration number increases, the ratio of high‐asymmetry structures in the training dataset increases. After several iterations of training with the generative model using the hybrid dataset, high‐asymmetry metamaterial structures with high‐FoM THz resonances can be generated by the well‐trained generative model.

### 2.2 Generative Diffusion Model for Inverse Design

**Figure 3:**
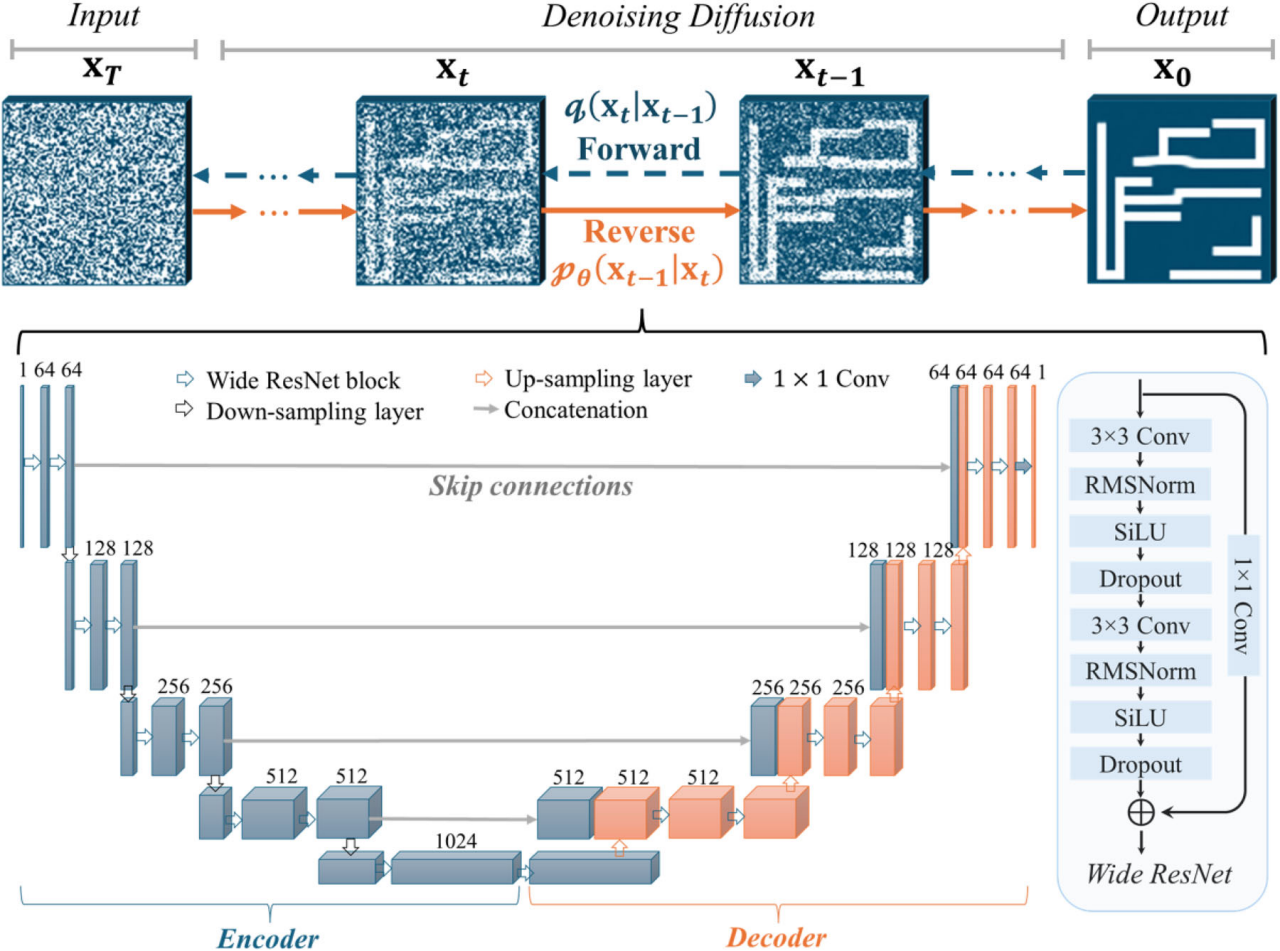
The architecture of the diffusion model for generating high‐asymmetry metamaterial structures. The diffusion model includes a forward diffusion process $q(x_{t}|x_{t-1})$ and a reverse diffusion process $p_{\theta }\left(x_{t-1}|x_{t}\right)$. Using the residual U‐Net with well‐trained parameters θ to estimate the noise image in each time step *t*, the structural image of high‐asymmetry metamaterial **x**_0_ can be reconstructed from the input random noise **x**_*T*_ after *T* steps of denoising diffusion.

Different from existing deep learning‐based inverse designs that use VAEs or GANs as generative models,^[^ ^50^, ^51^, ^52^, ^53^, ^54^, ^55^, ^56^ ^]^ we employ the diffusion model with superior generative capability to produce high‐asymmetry THz metamaterial structures. Compared to classical generative models, the diffusion model synthesizes images in higher diversity and quality with better stability in the training process.^[^ ^61^, ^62^ ^]^ The comparative study to evaluate the generative performance and computational costs of VAE, GAN, and the diffusion model can be found in Note S10, Figure S13, and Table S2, Supporting Information. As illustrated in **Figure** **3**, the diffusion model includes a forward diffusion process and a reverse diffusion process. In the forward diffusion process, given a metamaterial structural image from the training data distribution $x_{0}∼q\left(x_{0}\right)$, we add small Gaussian noise to the input structural image in *T* steps and obtain a sequence of noised structural images **x**_1_, ⋅⋅⋅,  **x**_*T*_. When *T* is sufficiently large, the structural image **x**_0_ gradually loses its distinguishable features as the step *t* increases. Under Markov assumption, the forward transition can be expressed as^[^ ^62^, ^63^ ^]^

(2) $$q\;\left(x_{t}|x_{t-1}\right)=N\left(x_{t};\sqrt{1-\beta_{t}}x_{t-1},\beta_{t}I\;\right)$$

where ${\left\{\beta_{t}\in \left(0,1\right)\right\}}_{t=1}^{T}$ is a variance schedule to ensure that the perturbed image **x**_*T*_ is nearly an isotropic Gaussian distribution, N(x;μ,σ) represents a normal distribution with mean μ and covariance σ generating the structural image **x**, and **I** is an identity matrix with the same dimension as the input image. Reversely, in the reverse diffusion process, the structural image can be reconstructed from an input Gaussian noise image $x_{T}∼N\left(0,I\;\right)$ following *T* reverse steps with $p(x_{t-1}|x_{t})$. An image‐to‐image translation network with the trainable parameters θ is trained to approximate each reverse step $p_{\theta }\left(x_{t-1}|x_{t}\right)$ via predicting the added Gaussian noise step by step. The reverse step is expressed as^[^ ^62^, ^63^ ^]^

(3) $$p_{\theta }\;\left(x_{t-1}|x_{t}\right)=N\left(x_{t-1};\mu_{\theta }\left(t,x_{t}\right),\Sigma_{\theta }\left(t,x_{t}\right)\;\right)$$

where **μ**_θ_(*t*,**x**_*t*_) and **Σ**_θ_(*t*,**x**_*t*_) represent the mean and covariance in the step *t*, respectively. Applying the reverse transition for all time steps, the data distribution becomes

(4) $$p_{\theta }\;\left(x_{0:T}\right)=p\left(x_{T}\right)\prod_{t=1}^{T}p_{\theta }\left(x_{t-1}|x_{t}\right)$$

With the known noise image **x**_*T*_ and the embedding at time step *t*, the network learns to reversely predict the noise and restore the structural image **x**_0_ after *T* steps.

The training of diffusion model is to find the reverse transitions that maximize the likelihood of the training data. Using the variational lower bound (VLB) combining with Kullback‐Leibler (KL) divergence, the loss function at each time step τ_*t*_ can be expressed as (See Note S3, Supporting Information)^[^ ^62^, ^63^, ^64^, ^65^ ^]^

(5) $$\tau_{t}=E_{t\in \left[1,T\right],x_{0},\varepsilon_{t}}\;\left[∥\varepsilon_{t}-\varepsilon_{\theta }\left(\sqrt{{\hat{\alpha }}_{t}}x_{0}+\sqrt{1-{\hat{\alpha }}_{t}}\varepsilon_{t},t\right){∥}^{2}\right]$$

where α_*t*_ =  1 − β_*t*_, ${\hat{\alpha }}_{t}=\prod_{i=1}^{t}\alpha_{i}$, E represents the expected value, ε_*t*_ is the real noise at time step *t*, and ε_θ_ is the predicted noise by the network at time step *t*. This effectively measures the distance between the real noise and the predicted noise, and then the network is trained to estimate the noise image at each time step and finally reconstruct the structural image. Using the well‐trained model, new structures with the features extracted from the provided dataset are generated by feeding random noise images into the trained reverse diffusion process (See Note S3 and Figure S4, Supporting Information).

As presented in Figure 3, the image‐to‐image translation network adopted to reconstruct the structure image step by step is a residual U‐shaped network (U‐Net), which consists of an encoder and a decoder with skip connections between them.^[^ ^66^ ^]^ The encoder includes four encoding modules, and the decoder includes four symmetrical decoding modules. Each encoding module has two Wide Residual Network (Wide ResNet) blocks and one down‐sampling layer.^[^ ^67^ ^]^ Each decoding module has one up‐sampling layer and two Wide ResNet blocks. The architecture of the Wide ResNet block includes two convolutional layers followed by root mean square layer normalization (RMSNorm), Sigmoid linear unit (SiLU) activation, and dropout layer (Dropout), together with the residual connection. The details of the network architecture can be found in Note S4 and Table S1, Supporting Information. To specify the diffusion time step *t* in the network, the sinusoidal position embedding is incorporated into each residual block.

### 2.3 Classical THz Metamaterial Structure Collection

To train the active learning‐augmented diffusion model, we collect a small set of classical THz metamaterial structures demonstrated in existing studies as the initial training dataset. The details of these structures and their corresponding transmissive properties obtained via simulation are presented in **Table** **2**. In the simulation, we set the substrate material as silicon dioxide (SiO_2_) and the metal resonator material as 200 nm thick aluminum (Al). The unit‐cell period is 100 × 100 µm^2^. The incident and detected THz wave are both *x*‐polarized. We define the asymmetry (φ) of each unit‐cell structure as $\varphi =\frac{\sum_{j=1}^{J}\sum_{k=1}^{K}\left(x_{j,k}\neq {\bar{x}}_{j,k}\right)}{J\times K}$ , where $\bar{x}$ with a dimension of *J* × *K* represents the mirrored image of **x** with its rows flipped in the *x* axis. *j* and *k* are the indices of the dimension of each image. All the asymmetry factors of the classical structures shown in Table 2 are below 0.15. As the resonant frequencies of these structures are slightly different, besides Q and FoM, we use an improved figure of merit (IFoM) that does not consider the specific resonant frequency to fairly evaluate the resonance performance, which is defined as *IFoM*  = *I*/*FWHM* . It is important to note that IFoM is not introduced as an optimization objective in our design process, but rather as a supplementary metric to neutralize the influence of resonance frequency in performance evaluation. Further demonstrations of using IFoM as the selection criterion in the second‐step annotation can be found in Note S14 and Figure S19, Supporting Information. The maximum and average Q, FoM, and IFoM of each type of structures according to the simulated transmissions have been listed in Table 2. It can be observed that the maximum Q, FoM, and IFoM among these structures reach 18.16, 2.69, and 4.53, respectively. In total, 68 structural images consisting of 14 types of symmetric or low‐asymmetry THz metamaterial structures with various geometric parameters are fed into the diffusion model for the first iteration of training (See Note S5 and Figures S5, S6, Supporting Information). Besides, we have conducted physical analysis on representative structures corresponding to the classical resonance types, including Fano, BIC, EIT, and toroidal dipole, to confirm the claimed coupling phenomena, as presented in Note S12 and Figures S15–S18, Supporting Information. As the high‐performance THz resonances of these structures have been demonstrated in existing studies,^[^ ^14^, ^15^, ^16^, ^17^, ^18^, ^19^, ^20^, ^21^, ^22^, ^23^, ^24^, ^25^, ^26^, ^27^, ^28^, ^29^, ^30^, ^31^, ^32^, ^33^, ^34^ ^]^ the network will automatically extract their underlying features in the training process and then be guided to generate new structures with THz resonances in the sampling process.

**Table 2:** Classical symmetric or low‐asymmetry THz metamaterial structures and their corresponding transmissive properties.

Index	1	2	3	4	5	6	7	8	9	10	11	12	13	14
Structure 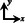	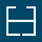	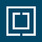	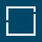	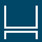	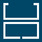	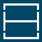	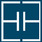	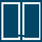	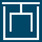	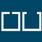	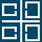	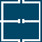	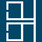	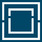
*T* ^a)^	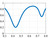	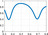	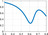	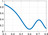	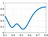	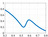	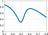	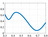	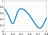	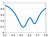	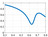	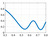	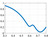	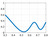
Principle	Fano	Fano/BIC	Fano/BIC	Fano/BIC	Fano	Toroidal dipole	Toroidal dipole/BIC	BIC	EIT	BIC	BIC	BIC	EIT	Fano
References.	[14, 15, 16]	[17, 18]	[20, 21, 22]	[23]	[15, 16]	[33]	[34]	[29]	[30]	[25, 26]	[27]	[28]	[31, 32]	[24]
φ ^b)^	0.04	0.02	0.01	0.14	0.13	0.00	0.00	0.01	0.14	0.10	0.00	0.00	0.00	0.01
Ave. *Q* ^c)^	4.08	2.30	4.18	2.85	3.33	2.30	2.75	4.92	3.04	7.72	7.01	5.36	**12.98**	3.23
Max. *Q*	9.96	4.67	14.08	3.95	4.43	2.91	3.35	7.00	6.71	15.55	12.87	13.10	**18.16**	4.77
Ave. *FoM* ^d)^	1.34	0.96	**1.40**	0.78	0.98	0.71	1.04	1.03	0.95	0.85	1.30	0.98	0.93	0.47
Max. *FoM*	**2.69**	2.22	2.47	0.93	1.67	0.72	1.45	1.87	1.59	1.51	2.60	1.76	1.12	0.72
Ave. *IFoM* ^e)^	**2.73**	1.78	2.51	1.39	2.04	1.32	2.15	2.30	1.63	1.47	2.09	1.63	1.38	0.80
Max. *IFoM*	4.43	3.85	**4.53**	1.63	3.36	1.42	3.11	3.12	2.41	2.90	4.14	2.49	1.54	1.11

^a)^ The horizontal and vertical axes of each curve represent frequency in THz and the transmission *T*, respectively;

^b)^ Here φ represents the average asymmetry factor of each type of structures in the dataset;

^c)^ *Q*  = *f_r_*/*FWHM* , where *f_r_* is the resonant frequency and *FWHM* measures the full width half maximum of the resonant peak;

^d)^ *FoM*  =  *Q* × *I*, where *I* is the contrast between the maximum and minimum values of each resonant peak;

^e)^ *IFoM*  = *I*/*FWHM*  and the unit is THz^−1^. It should be noted that these metrics of each resonance peak for every structure are calculated, and the maximum (Max.) and average (Ave.) values among these metrics are listed.

### 2.4 Numerical Demonstrations of Generated High‐Asymmetry Structures

**Figure 4:**
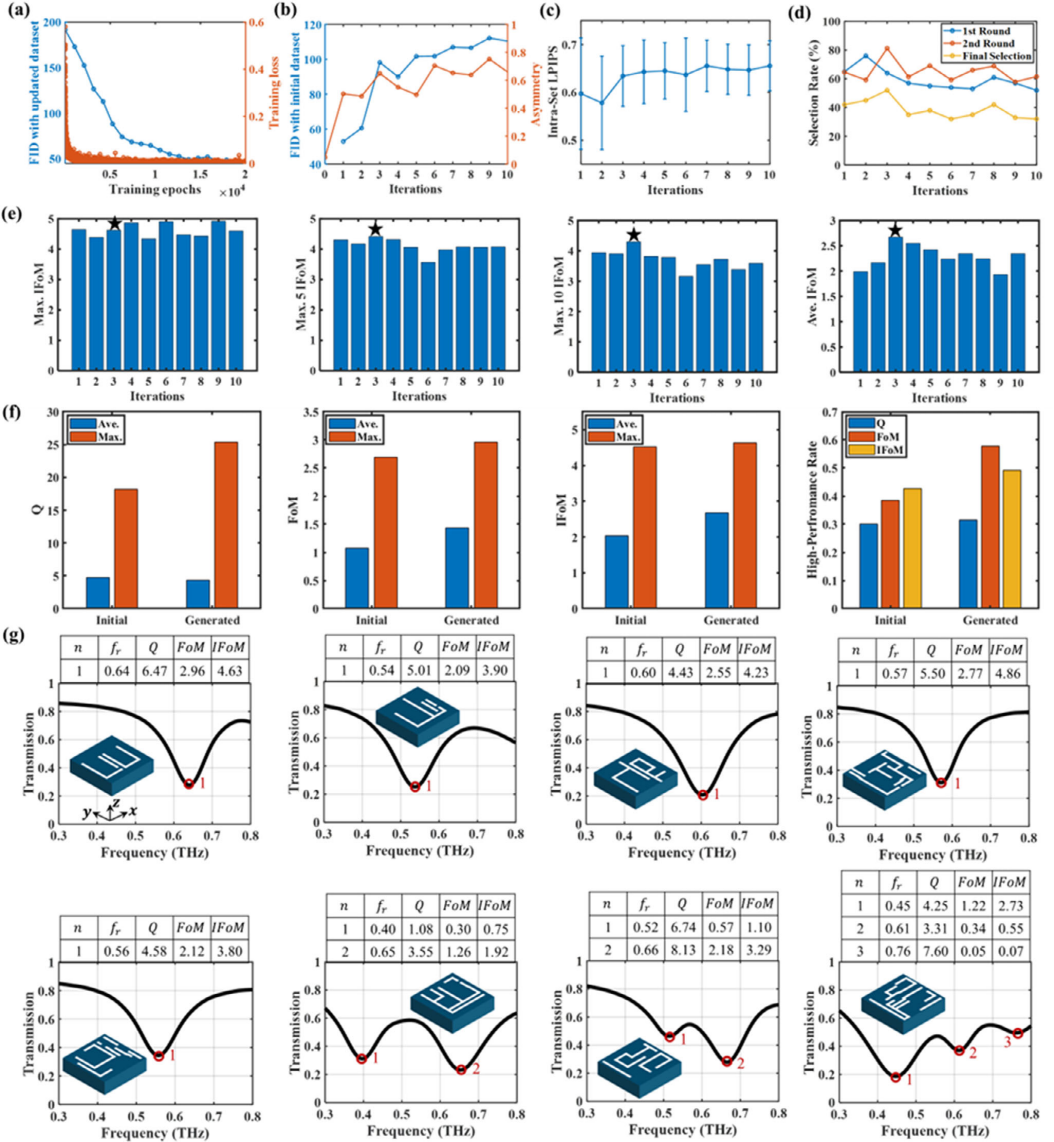
Illustration of the generated high‐asymmetry structures. a) The training loss and FID curves with increasing epochs in one single iteration, in which the FID measures the feature distance between the generated structures and the training dataset updated in each iteration. b) The FID score and average asymmetry of the generated structures with increasing iterations, in which the FID measures the feature distance between the generated structures and the initial training dataset containing only initial classical structures. c) The LPIPS curves of the generated structures with increasing iterations, in which LPIPS measures the intra‐set diversity of the generated structure set. d) The curves of selective rates when using the physics‐constrained labeling mechanism in the ten iterations. The final selection rate is the product of the 1^st^‐round and the 2^nd^‐round selection rates. e) The IFoM curves of the generated high‐asymmetry structures. f) Metrics comparison of the initially involved classical structures and the generated high‐asymmetry structures in the 3^rd^ iteration. g) Simulation results of eight examples of the generated high‐asymmetry structures, including single‐, double‐, and totally new triple‐resonance metamaterials.

The iteratively updated dataset that combines the initial low‐asymmetry structures and the selected high‐asymmetry structures is used for training the diffusion model in the active learning loops. We train the diffusion model for 10 iterations, and each iteration includes 20,000 training epochs. After each iteration, we use the trained model to generate 100 new high‐asymmetry structures and verify their transmissive properties via simulations, resulting in 1,000 forward simulations in total. **Figure** **4**a shows one example of the curves of training loss defined in Equation (5) and Fréchet inception distance (FID) between the generated structures and the initial structures with training epochs in one iteration.^[^ ^68^ ^]^ Both the loss and FID score decrease dramatically at the beginning and then tend to converge, demonstrating the effective training of the diffusion model in a single iteration. It should be noted that FID scores measure the feature distance between the generated structure and the training dataset across training epochs in each iteration, which is updated and expanded as the iteration increases due to the proposed active learning mechanism that adds new high‐asymmetry structures with high FoMs into the training dataset after each iteration of training. All the loss curves and FID curves with the training epochs in the ten iterations as well as the detailed analysis can be found in Note S2 and Figures S2, S3, Supporting Information.

To prove the effectiveness of the proposed active learning mechanism, we further calculate the average asymmetry (φ) of the initial classical structures and the generated structures in each iteration as well as the FID between the initial classical structures and the generated structures across the ten iterations. As shown in Figure 4b, the average asymmetry of the generated structures increases with the number of iterations. Compared to the initial dataset (Iteration 0), the generated structures exhibit significantly higher levels of asymmetry. Also, the FID score rises with the iteration. Here, different from the FID calculations in Figure 4a, the reference dataset in each iteration is always the initial training dataset containing only the classical structures. Therefore, the increasing trend of the FID score in Figure 4b reflects the generalization capability of the proposed model on generating new structures with the rising asymmetry that are far away from the initial provided dataset. However, the growth rates of the asymmetry and FID score slow down after the 3^rd^ iteration, demonstrating the converged learning capability of the diffusion model training with a small dataset. Although the resonance performance of a unit‐cell structure is not always related to its asymmetry as the specific geometry pattern should be considered, the upward trend in the average asymmetry and FID score indicates the increasing deviation of the generated structures from the initial dataset and the growing potential of the generative model for producing new complicated high‐asymmetry designs. Besides the FID score that measures the extra‐set diversity of the generated structures against the classical structures, we calculate the learned perceptual image patch similarity (LPIPS) of the generated structures across the ten iterations to demonstrate the intra‐set diversity of the generated structures. It measures perceptual similarity between random pairs of the generated samples and a higher LPIPS represents a better intra‐set diversity of the generated structure set.^[^ ^69^ ^]^ The LPIPS curve across the ten iterations is presented in Figure R7c. Similarly to the FID scores shown in Figure 4b, the LPIPS rises as the iteration increases and then tends to be steady, proving the model generalizability convergence. Figure 4d presents the selection rates of the first‐step data query and the second‐step annotation as well as the final selection rate in each iteration. The initial increase in the selection rates is due to the rising number of high‐asymmetry, high‐FoM structures in early iterations. The subsequent decline results from the saturation of the model's generalization capability. Trained on only 68 structures from 14 classical types, the generative model is limited by the initial dataset's diversity. Although the proposed active learning mechanism augments the data, new samples remain confined to the original data space. By the third iteration, the model's ability to generate new high‐FoM designs plateaus, leading to a decrease in selection rates.

To evaluate the resonance performance of the generated high‐asymmetry structures, we calculate the maximum IFoM (Max. IFoM), average of the top ten maximum IFoMs (Max. 10 IFoM), average of the top five maximum IFoMs (Max. 5 IFoM), and the average IFoM (Ave. IFoM), in each iteration and show them in Figure 4e. These metrics tend to increase and the overall resonance performance of generated structures in the 3^rd^ iteration reaches the best. Figure 4f compares the performance of the classical structures in the initial dataset and the high‐asymmetry structures generated in the 3^rd^ iteration. It can be found that the resonance metrics, including Q, FoM, and IFoM, of the generated high‐asymmetry structures outperform those of the initial training dataset, demonstrating that the generated structures break the limits of classical structures. Besides, the high‐metrics rates of the initial dataset and the generated structures are separately obtained via dividing the number of structures whose metrics are greater than the average by the total number. All the high‐Q, FoM, and IFoM rates of the generated structures are higher than those of the initially involved classical structures. Figure 4g shows eight examples of the generated high‐asymmetry structures and their simulated transmissions as well as the evaluation metrics, including single‐, double‐, and triple‐resonance structures. It should be noted that the classical structures adopted in the initial training dataset only have single and double resonance, while the generated high‐asymmetry structures not only have single and double resonances but also produce unseen triple resonances. The occasional appearance of triple‐resonance structures shows that our framework can discover complex designs beyond the simple cases used for training. Although such events are rare, their stable and reproducible occurrence highlights the model's potential to uncover advanced structures that are otherwise difficult to obtain. More triple‐resonance structures and related analysis can be found in Note S6, Figure S7, and Table S2, Supporting Information.

### 2.5 Experimental Demonstrations and Performance Comparison

**Figure 5:**
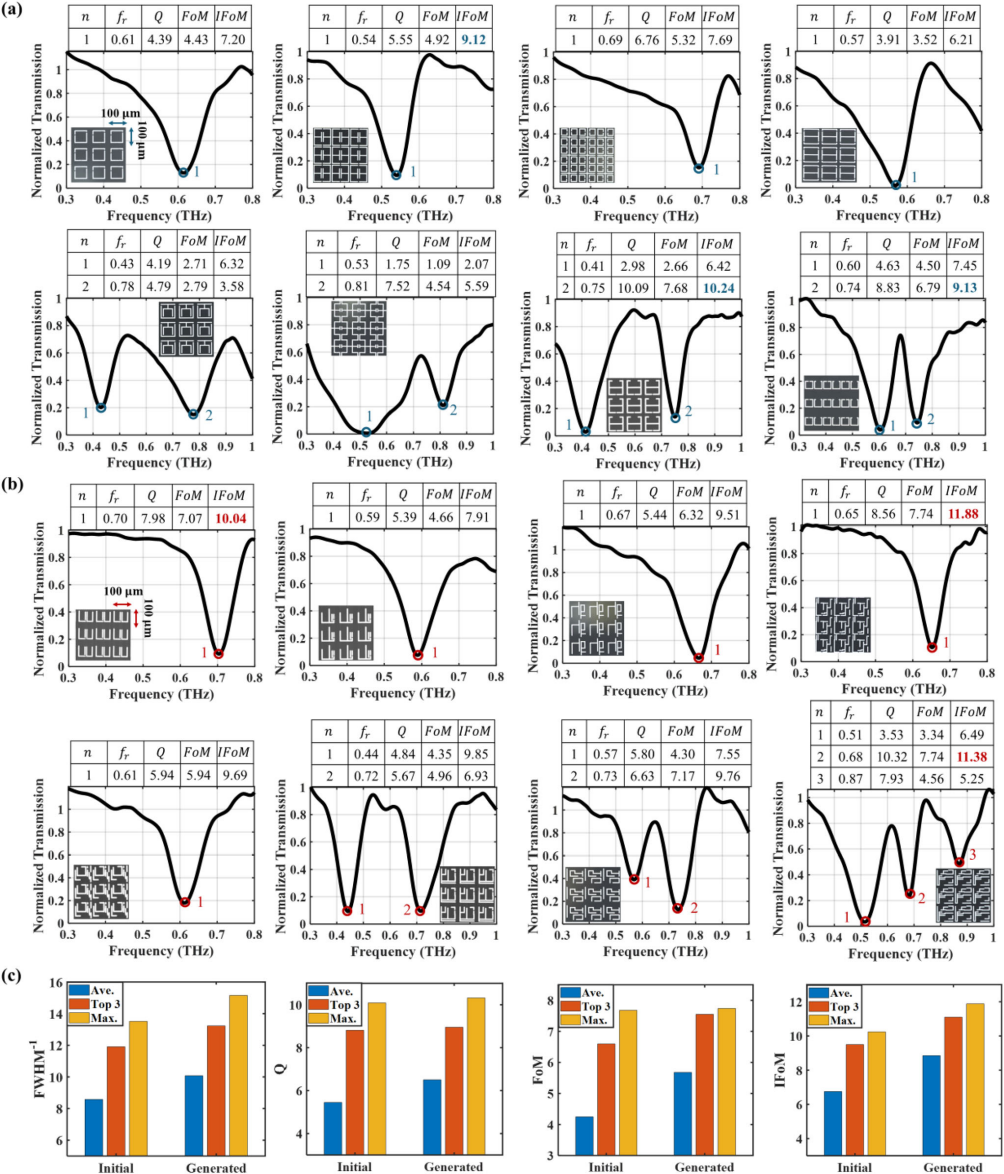
Experimental result comparison of the involved traditional structures and the generated high‐asymmetry structures. a) The measured transmission and metrics of traditional THz metamaterial structures. b) The measured transmission and metrics of the generated structures. c) Metrics comparison between the classical structures in the initial dataset and the generated structures involved in the experimental characterization. The unit of FWHM^−1^ is THz^−1^.

We fabricated eight high‐asymmetry metamaterial structures shown in Figure 4g and eight classical structures selected from Table 2 for experimental characterization. **Figure** **5** shows the microscopic images of partial arrays consisting of unit‐cell structures and the measured transmissions as well as the evaluation metrics of each THz resonance. Each array includes 100×100 unit cells with a periodicity of 100×100 µm^2^. The original measured transmissions of the eight classical structures and the eight generated structures can be found in Figure S11, Supporting Information. To fairly compare their resonance metrics, the measured transmission through the metadevice is normalized against the transmission through a bare quartz substrate. Figure 5a presents the normalized transmissions of the classical structures, in which single and double resonances occur. The top three IFoMs of these structures are 10.24, 9.13, and 9.12. Figure 5b shows the results of the generated high‐asymmetry structures that exhibit not only single and double resonance but also the newly generated triple‐resonance phenomena. The top three IFoMs of these high‐asymmetry structures are as high as 11.88, 11.38, and 10.04, which outperform the classical structures. To quantitatively evaluate the resonance performance, Figure 5c compares the average value (Ave.), the maximum value (Max.), and the average of the top three maximum values (Top 3) for the FWHM^−1^, Q, FoM, and IFoM of the fabricated classical structures selected from initial dataset and the high‐asymmetry structures generated by the proposed scheme. It can be observed that all the metrics of the generated structures surpass those of classical structures, with notable improvements in average FWHM^−1^ (17.48%), Q (19.27%), FoM (33.65%), and IFoM (31.11%), demonstrating the superior resonance performance of the high‐asymmetry structures designed by the proposed generative model.

## 3 Conclusion

We propose a novel design scheme for high‐asymmetry THz metamaterials via an active learning‐augmented diffusion model that aims at addressing the limitations of conventional rational design and existing deep learning‐based design. Particularly, conventional rational design approaches for THz metamaterial structures highly depend on human expertise and iterative trial‐and‐error optimization, and are inherently constrained by human intuition and creativity, leaving the design of high‐asymmetry structures extremely challenging. Although recent deep learning‐based methods accelerate and automate the design process, they are limited by the high requirements for diverse and large datasets and the heavy computational costs, especially for asymmetric structures with high geometric complexity. In contrast, our proposed scheme learns the underlying features from a small set of classical low‐asymmetry structures with prior knowledge, which mirrors how human designers draw inspiration from literature reviews in the rational design process. Different from existing studies that involve VAEs or GANs as generative models for metamaterial design, this work adopts the advanced diffusion model with a stepwise denoising process to generate high‐asymmetry structures in high quality and diversity. To further overcome the dataset limitation, a physics‐constrained active learning framework with a two‐step labeling mechanism is developed to iteratively augment the training dataset and improve the THz resonance performance of the generated high‐asymmetry structures.

The numerical and experimental results showed higher THz resonance metrics of the generated high‐asymmetry structures compared to those of classical structures in the initial training dataset. In the experimental demonstration, the high‐asymmetry metamaterials achieved 17.48%, 19.27%, 33.65%, and 31.11% improvements of the average FWHM^−1^, Q, FoM, and IFoM, respectively, and all the maximum FWHM^−1^, Q, FoM, and IFoM outperformed those of the classical structures. More comparison with existing studies on FoM metrics can be found in Note S13 and Table S4, Supporting Information. Besides, the generated structures exhibited novel triple‐resonance phenomena, which were absent in the initial training dataset containing only single‐ or double‐resonance structures. The occasional but stable appearance of triple‐resonance structures demonstrates that our framework can reproducibly discover complex and hard‐to‐obtain designs beyond the simple cases used for training. More importantly, only 68 classical structures were used for initially training the generative model, demonstrating highly enhanced efficiency compared to existing deep learning‐based inverse design that required thousands of training samples. These findings verify the high‐asymmetry metamaterial structures to be a promising alternative solution to high‐FoM THz resonance, broadening their potential applications in high‐sensitivity THz metadevices (See Note S9 and Figure S12, Supporting Information). Furthermore, the proposed scheme that overcomes the data limitations not only provides an efficient and effective method to design complicated metamaterial structures but also holds great potential for discovering new metamaterials that break existing limitations. Even when the design target of the metamaterial structure is changed, the proposed scheme can be well applied via modifying the small classical dataset and the selection critics in the active learning process (See Note S11 and Figure S14, Supporting Information). Despite these promising results, the generalization capability of the proposed model is limited by the diversity of the initial dataset, and the lack of explicit physical priors hinders interpretability and control over specific resonance mechanisms. Our future work will address these limitations by expanding the initial dataset with more diverse classical structures and integrating more physical insights into the generative process.

## 4 Experimental Section

### Implementation of Active Learning‐Augmented Diffusion Model

The proposed active learning‐augmented diffusion model was implemented on the open‐source machine learning platform PyTorch.^[^ ^70^ ^]^ The iterative process, including 1) training the diffusion model, 2) calling the simulator for each generated structure, 3) obtaining *n_eff_* for the 1^st^ step of selection, 3) calculating the resonance metrics for the 2^nd^ step of selection, and 4) adding the finally selected structures into training dataset for the next loop (See Note S2 and Figure S1, Supporting Information), was integrated into an automatic mode using Python language combined with MATLAB program. The initial training dataset included 68 classical structures collected from existing studies. After ten iterations, the amount of data samples increased to 454. In the learning process of the diffusion model, the following parameters were set as: the structural image dimension 100×100, the batch size 16, the learning rate 2×10^−5^, the training epochs in each iteration 2×10^4^, the total number of iterations 10, the number of samplings in each iteration 100, the time steps in the training of diffusion model 1000, and the time steps in the sampling of diffusion model 250. The whole framework was implemented on a workstation with an NVIDIA GeForce RTX 4090 GPU.

### Numerical Simulations

The numerical simulations were performed using a 3D finite‐difference time‐domain (FDTD) program (Lumerical FDTD). When importing the generated structural images into the simulator, the threshold as 0.5 was set to ensure the structural image to be binary. In the simulation, the complex refractive indices of Al and SiO_2_ was used from CRC et al. and Palik et al., respectively. The thickness of Al was set to 200 µm. The thickness of SiO_2_ was set as infinite. The simulation was performed on a unit cell with periodic conditions in *x* and *y* directions and the perfect matching layer (PML) boundary conditions in *z* direction. The *x*‐polarized THz wave was incident into the device along *z* axis and the co‐polarized transmission was detected. All the simulated results were extracted using the MATLAB program.

### Sample Fabrication

The complete fabrication procedure for the samples comprises the subsequent steps. (1) Quartz substrate cleaning: The quartz substrate underwent an immersion process using acetone and IPA separately. It was then thoroughly rinsed with deionized water and dried with a nitrogen gun to ensure a clean surface. (2) Photoresist coating: The photoresist AZ1512 was spin‐coated on the surface of the quartz substrate, spun at 6000 rpm for 90 s. After coating, it was baked at 105°C for 1 min. (3) Patterning and developing: The Laser Writer – Hiedelberg DWL66+ (focus: 25%; intensity: 100%; laser power: 95 mW; filter: 1%) was used for patterning. After the patterning, the device was baked at 115°C for 1 min. Then the device was immersed in the developer MF319 for 1 min, rinsed with deionized water, and dried with nitrogen. The exposure effects of the device were checked under an optical microscope. (4) Metal deposition: Using the E‐Beam Evaporator – AJA UHV, Ti with a thickness of 10 nm was first deposited as an adhesion layer and 200 nm thick Al was then deposited onto the sample. (5) Lift‐off: The device was immersed in acetone and then the ultrasonic cleaning process removes the photoresist under heated conditions. The illustration of the fabrication process can be found in Note S7 and Figure S8, Supporting Information. In total, eight samples of the classical low‐asymmetry structures and eight samples of the generated high‐asymmetry structures were fabricated and stored in a dry and sealed environment prior to use. The microscopic images of the sixteen fabricated samples can be found in Note S7 and Figure S9, Supporting Information.

### THz Characterization

A commercial photoconductive antenna‐based THz spectroscopy setup placed inside a nitrogen gas filled chamber was used for the metadevice characterization (See Note S8s and Figure S10, Supporting Information). Each sample was mounted on a chip carrier with a circular aperture. The incoming THz wave was incident normally on the sample and the transmitted co‐polarized signal was measured.

## Conflict of Interest

The authors declare no conflict of interest.

## Supporting information



Supporting Information

## Data Availability

All the datasets and implementation codes that support the findings of this study will be released at https://github.com/Qiqi‐Dai upon publication.
