# Supplementary material for: High‐Asymmetry Metasurface: A New Solution for Terahertz Resonance via Active Learning‐Augmented Diffusion Model
Source: Adv Sci (Weinh). 2025 Sep 23;13(2):e08610. doi: 10.1002/advs.202508610 (PMC12786302; doi:10.1002/advs.202508610)
Supplement: Supplementary file 1 — Supporting Information [file ADVS-13-e08610-s001.pdf]

Supplementary Information for

High-Asymmetry Metasurface: A New Solution for Terahertz Resonance via Active Learning-Augmented Diffusion Model

Qiqi Dai[1,2], Yinpeng Wang[1,2], Cheng Xu[1,2], Dongxiao Li[1,2], Prakash Pitchappa[3], Thomas Caiwei Tan[4], Ranjan Singh[4,5,6,] and Chengkuo Lee[1,2,6,*]*

[1]Department of Electrical & Computer Engineering, National University of Singapore, 4 Engineering Drive 3, Singapore 117576, Singapore.

[2]Center for Intelligent Sensors and MEMS (CISM), National University of Singapore, 5 Engineering Drive 1, Singapore 117608, Singapore.

[3]Institute of Microelectronics (IME), Agency for Science, Technology and Research (A*STAR), 2 Fusionopolis Way, Innovis #08-02, Singapore 138634, Republic of Singapore.

[4]Division of Physics and Applied Physics, School of Physical and Mathematical Sciences, Nanyang Technological University, Singapore 637371, Singapore.

[5]Department of Electrical Engineering, University of Notre Dame, Notre Dame, IN, 46556 USA.

[6]National Centre for Advanced Integrated Photonics (NCAIP), Singapore 639798, Singapore.

[*]E-mail: rsingh3@nd.edu; elelc@nus.edu.sg

Table of Contents

Supplementary Note 1: Background of THz Metamaterial Development.......................... 2
Supplementary Note 2: Implementation of Physics-Constrained Active Learning Loops 7
Supplementary Note 3: Algorithms of Diffusion Model. 11
Supplementary Note 4: Network Architecture. 13
Supplementary Note 5: Classical Structures in Initial Training Dataset. 15
Supplementary Note 6: More Trip-Resonance Structures. 21
Supplementary Note 7: Microfabrication Process and Results. 23
Supplementary Note 8: THz Measurement Setup and Original Measurement Data...... 25
Supplementary Note 9: Investigations on Potential Sensing Applications. 27
Supplementary Note 10: Comparative Study of Generative Models. 29
Supplementary Note 11: Applicability of the Proposed Scheme to Different Target...... 31
Supplementary Note 12: Physical Analysis on Classical Structures 33
Supplementary Note 13: Resonance Metric Comparison with Existing Works 41
Supplementary Note 14: Difference between FoM and IFoM 42
Supplementary References 46

Supplementary Note 1: Background of THz Metamaterial Development

Terahertz (THz) metamaterials are artificially engineered structures designed to control and manipulate electromagnetic waves in the frequency range of 0.1–10 THz. THz metamaterials bridge the "THz gap" and enable precise control over THz waves for a wide range of groundbreaking applications, including non-invasive medical imaging,[1-3] chemical and biological sensing,[4-6] high-speed wireless communication,[7,8] security screening,[9,10] and advanced spectroscopy.[11,12]

Over the years, a diverse set of THz metamaterials with carefully designed subwavelength patterns have been developed to achieve THz resonances that facilitate strong light-matter interactions for enabling high sensitivity. The most popular structures were split ring resonators (SRRs) proposed by Pendry et al and then expanded to THz devices, in which the SRRs exhibited a strong magnetic response with negative permeability for achieving a negative index of refraction at the inductive-capacitive (LC) resonance mode.[13-15] To further improve the resonance performance, SRRs with various modified forms were investigated for satisfying unique properties, such as Fano resonance, electromagnetically induced transparency (EIT), bound states in the continuum (BIC), and toroidal dipole.[16-35] For instance, two individual asymmetric D-split resonators (ADSRs) were used as unit cells to suppress radiation losses and achieve THz resonance with high quality factor (Q).[16,17] The coupling coefficient between two opposite nested metallic SRRs was optimized to satisfy the Friedrich-Wintgen (resonance-trapped) BIC with normal THz incidence.[18-20] The SSR with dual capacitive gaps at asymmetric positions, enabling two unequal metallic wires to form an asymmetric resonator, was developed to excite Fano resonance with a figure of merit (FoM) that considers both Q and resonance intensity.[21-23] Side coupled configuration with the resonating arms flipped was further designed to raise a symmetry-protected BIC for the coupling of Fano resonance to the first-order lattice mode of the metamaterial array, leading to the improvements of both the Q and FoM.[24] The coupling of two concentric SRRs with different gap positions was studied to build an asymmetric metamaterial structure to realize THz Fano resonances.[25] Adjacent four square SSRs with asymmetric gaps formed one superlattice to achieve quasi-BICs for high-Q THz metasurface.[26,27] Four SRRs with asymmetric dimensions were distributed around the cut-wire to enhance the linewidth of quasi-BIC resonance.[28] T-shaped resonators and metallic loops between the adjacent unit cells were carefully designed to compose the resonator unit, supporting Friedrich-Wintgen BIC without breaking the mirror symmetry via tuning the coupling of the LC mode and dipole mode resonances.[29] Two symmetry-broken rectangular

SRRs by shifting one gap off the center were mirror-arranged to enable the occurrence of one trapped mode in addition to an octupolar mode leading to two distinct high-Q resonances.[30] Multi-band EIT effect of THz metamaterial was formed by two sub-resonators, a fork-shaped resonator and a U-shaped SRR, which corresponded to two different "big-bright" resonance modes.[31] The EIT analog in THz metamaterial was also excited via the strong conductive coupling between two orthogonally twisted SRRs.[32,33] Sharp toroidal dipolar response by symmetrically moving the pair of capacitive gaps toward central branch of SRRs was demonstrated.[34] Two E-shaped SRRs were mirror-located in the unit cell to form toroidal dipole metasurface driven by BIC for optimizing THz resonance quality.[35]

To expand functionalities of passive devices, active THz metadevices have gained increasing attention to realize dynamic modulation of THz electromagnetic waves.[36] One popular solution for tuning THz metadevices was integrating materials with active properties. The active materials were typically semiconductors,[15,37,38] liquid crystals,[39,40] phase change materials,[41,42] and 2D materials.[43,44] They can be integrated into the substrate, surrounding medium, or resonator of THz metamaterials, with their properties electrically, optically, thermally or magnetically tunable. Another important tuning mechanism was reconfiguring geometric structures via microelectromechanical systems (MEMS). Due to the broadband response, high reconfigurability, reduced material dependence, and lower energy consumption, a wide range of MEMS-actuated THz devices have been reported.[45] The predominant designs involved regulating the deformation of biomaterial microcantilevers in THz metadevices through electrostatic actuation or thermal/electrothermal configuration to enable various functionalities, such as frequency agile, bandwidth control, polarization control, phase transition, wavefront deflection, cloaking, and holograms.[46-58] For example, the bimorph cantilevers of electric split-ring resonator (eSRR) were dynamically adjusted via applying direct current voltage between the released cantilevers and the silicon substrate to achieve a tunable electromagnetic response including multiband resonance frequency operation and polarization dependent tunability.[47] Active phase diagram was determined by the suspension angle of the individual bimorph cantilever in THz metamaterials controlled through electrostatic actuation, enabling the applications in polarization control, wavefront deflection, and holograms.[53,54] Resonant frequency tuning and amplitude modification of THz waves were achieved through the integration of a cut-wire microcantilever electromechanically reconfigured via current stimulus on a photoresponsive ion-irradiated silicon substrate with optical stimulus.[58]

Among the above THz metamaterials based on rational design, symmetric or low-asymmetry geometry patterns were adopted as unit-cell structures. On the one hand, in the rational design process, repetitive trial-and-error optimizations are usually required to satisfy the desired specifications, remaining it time- and labor-consuming to find the optimal structures and geometric parameters. On the other hand, due to the limitations of human imagination, the design of irregular high-asymmetry structures with complicated geometry patterns is challenging via traditional rational design.

To solve the limitations of rational design, deep learning-based techniques have been recently developed to design metamaterial structures.[59-62] The advantages of deep learning applied to metamaterial design lie in the strong learning capability and generalizability. Once a set of training samples are fitted into a deep neural network, the network can be trained to learn the features of the data samples and then provides similar outputs or even results beyond the performance of the training data. Automatic and rapid design for various metamaterial structures can be realized using the well-trained network. Existing metamaterial design based on deep learning includes forward design and inverse design. The forward design builds the mapping relationship from metamaterial structures to the corresponding electromagnetic response, such as transmissive/reflective phase and amplitude, while the inverse design maps the input desired response to the corresponding metamaterial structure using neural networks.[61] The geometry parameters of a specific metamaterial structure can be optimized using 1D neural networks, which often combines an inverse network and a forward network to enhance the design accuracy.[63-68] Although 1D networks had relatively low computational costs, their limited degrees of freedom (DoFs) restricted the design scope to one specific structure. For example, the lengths and spacer thickness of top and bottom U-shaped SRRs as well as the twisted angle were optimized using a bidirectional neural network to enhance the chiral response;[63] eight geometry parameters of H-formed plasmonic nanostructures were designed via a bidirectional neural network.[64] Therefore, it is highly challenging to design high-asymmetry structures via 1D deep learning models.

To expand DoF of metamaterial design, 2D neural networks, including 2D convolutional neural networks (CNNs), generative adversarial networks (GANs), and variational auto-encoders (VAEs), have been increasingly explored for designing 2D geometric patterns of metamaterial structures.[69-78] 2D CNNs were usually used for forward design, in which they were trained using a set of structural images as inputs and the corresponding electromagnetic properties as outputs. Using the well-trained CNNs, the responses of new structures were predicted in real

time and the optimal structure could be quickly selected.[69-71] However, one challenge in the implementation process was the requirement of a large dataset for training the 2D CNNs, such as the collection of seventy thousand geometry patterns of meta-atoms.[69] To reduce this workload, transfer learning was introduced to pre-train the network using an available large dataset and then fine-tune the pre-trained network using the collected metamaterial structure set, while twenty thousand samples were still employed for the fine-tuning.[70] As for 2D inverse design that intuitively obtained the geometric patterns from the desired response, 2D generative models, such as GANs and VAEs, were developed using the desired properties as the input conditions and the corresponding geometric patterns as the output images.[72-78] Similarly, they require a large amount of training data samples. To reduce the design complexity, the geometry pattern of a unit cell was designed with twofold symmetry, while over twenty thousand sets of samples were still used for the training.[74] Another method for reducing the workload of data collection was limiting the structures to several types of geometric shapes, such as cross, split ring, and H-shape, however, the newly generated structures were limited in these provided shapes as well.[76] To enlarge the dataset size, cyclic iteration was used to add the generated structures into the original dataset to increase the amount of training data samples in the iterative training process. Nevertheless, due to the purely data-driven characteristics of both the forward selection and inverse design process, the finally used dataset size yet reached over fifty thousand.[78] As the DoF and complexity of high-asymmetry metamaterials largely increase, the design for their geometry patterns using existing 2D deep learning-based methods particularly suffer from severe computational costs.

This work aims at addressing the challenges of traditional rational and existing deep learning-based design methods and exploring effective and efficient schemes for designing THz high-asymmetry metamaterials. A novel scheme based on an active learning-augmented diffusion model is proposed to design the structures of high-asymmetry metamaterials with high-FoM THz resonance. Only a small set of classical low-asymmetry structures obtained from literature review are required for training the generative model. To augment the dataset, we design a physics-constrained active learning framework with a two-step labeling mechanism to iteratively select the generated high-asymmetry structures as new training data. Instead of classical VAEs or GANs for inverse design, the advanced diffusion model is used as the generative model to generate high-asymmetry structures in high quality and diversity. The numerical and experimental results demonstrate the superior THz resonance of the generated high-asymmetry structures over classical low-asymmetry ones and reveal new triple-resonance phenomena. This proves the high-asymmetry metamaterial structures to be a promising

alternative solution to high-FoM THz resonance and establishes the proposed scheme as a powerful method for designing complex metamaterial structures, with great potential for discovering new metamaterials that break existing limitations.

Supplementary Note 2: Implementation of Physics-Constrained Active Learning Loops

Figure S1 shows the flowchart of implementing the proposed physics-constrained active learning framework for generating high-asymmetry structures with high-FoM THz resonance. The loop with black arrows represents the implementation process of the 1st iteration of training, in which the training dataset only involves classical low-asymmetry structures with THz resonance collected from existing studies. The trained diffusion model generates new structures from the input random noise images in the sampling process. After that, the FDTD simulator is used to calculate the real part of the effective refractive index $Re(n_{eff})$ for the generated new structure in order to select the valuable structures with potential THz resonance, following which the FoMs of the selected structures are computed to finally screen out the structures with high-FoM resonance. Then the loops with blue arrows represent the 2nd to nth iterations of training, in which the training dataset uses the hybrid set of the initial low-asymmetry structures and the selected high-asymmetry structures. It should be noted that only the high-asymmetry structures after the two steps of selections are combined into the training dataset, guiding the diffusion model to generate new structures with high-FoM THz resonance.

The curves of training loss and Fréchet inception distance (FID) with training epochs in each single iteration are plotted in **Figure S2**. The FID measures the distance between feature distributions of the training dataset and the generated structures in one single iteration, including 20,000 training epochs. It should be noted that the training dataset in each iteration is different as we update the training dataset via adding selected high-asymmetry, high-FoM structures into it. To further clarify the FID calculation, we define the training epoch in each iteration as $m \in [1, M]$ $(M = 20{,}000)$ and the iteration index as $n \in [1, N]$ $(N = 10)$. The FID score in each training epoch can be expressed as Equation S1. $\mu_{r_{n,m}}$ and $\mu_{g_{n,m}}$ represent means of real image features and the generated image features, respectively, in the mth training epoch of the nth iteration. $\sigma_{r_{n,m}}$ and $\sigma_{g_{n,m}}$ represent covariances of real image features and generated image features, respectively, in the mth training epoch of the nth iteration. As shown in Figure S2, the decreasing trend in each iteration demonstrates that the diffusion model is well trained and fitted with the updated training dataset, as the feature distribution of the generated structures becomes closer to that of the provided training dataset and gradually converges as the training epoch increases. To visually demonstrate this trend, **Figure S3**a shows the imaging results of 25 generated samples with an increasing training epoch in the first iteration, in which the training dataset includes only classical low-asymmetry structures. The decreased distance between the

distributions of generated samples and the training dataset demonstrates good convergence of the diffusion model in a single iteration.

After each iteration of training, we use the well-trained model with the lowest FID to generate 100 new structures and then calculate the FID between the generated structures in each iteration and the fixed initial training dataset that only includes the classical low-asymmetry structures. The expression of FID score in each iteration can be expressed as Equation S2. μ_{r_0} and σ_{r_0} represent the mean and covariance of the real image features, respectively, using the fixed initial training dataset. As shown in Figure 4b of the manuscript, as the iteration n increases and the asymmetry of the training dataset increases, the model is capable of generating higher-asymmetry structures that are further away from the distribution of the initial classical structures, resulting in the increased FID score. Figure S3b shows 25 generated samples in each iteration. We can visually observe that the complexity of the structures is rapidly increasing in the first three iterations and then slowly increasing in the last seven iterations, which is consistent with the FID trend shown in Figure 4b of the manuscript.

$$\mathrm{FID}_{n,m} = \left\|\mu_{r_{n,m}} - \mu_{g_{n,m}}\right\|^2 + \mathrm{Tr}\left(\sigma_{r_{n,m}} + \sigma_{g_{n,m}} - 2\left(\sigma_{r_{n,m}}\sigma_{g_{n,m}}\right)^{1/2}\right) \quad (S1)$$

$$\mathrm{FID}_n = \left\|\mu_{r_0} - \mu_{g_n}\right\|^2 + \mathrm{Tr}\left(\sigma_{r_0} + \sigma_{g_n} - 2\left(\sigma_{r_0}\sigma_{g_n}\right)^{1/2}\right) \quad (S2)$$

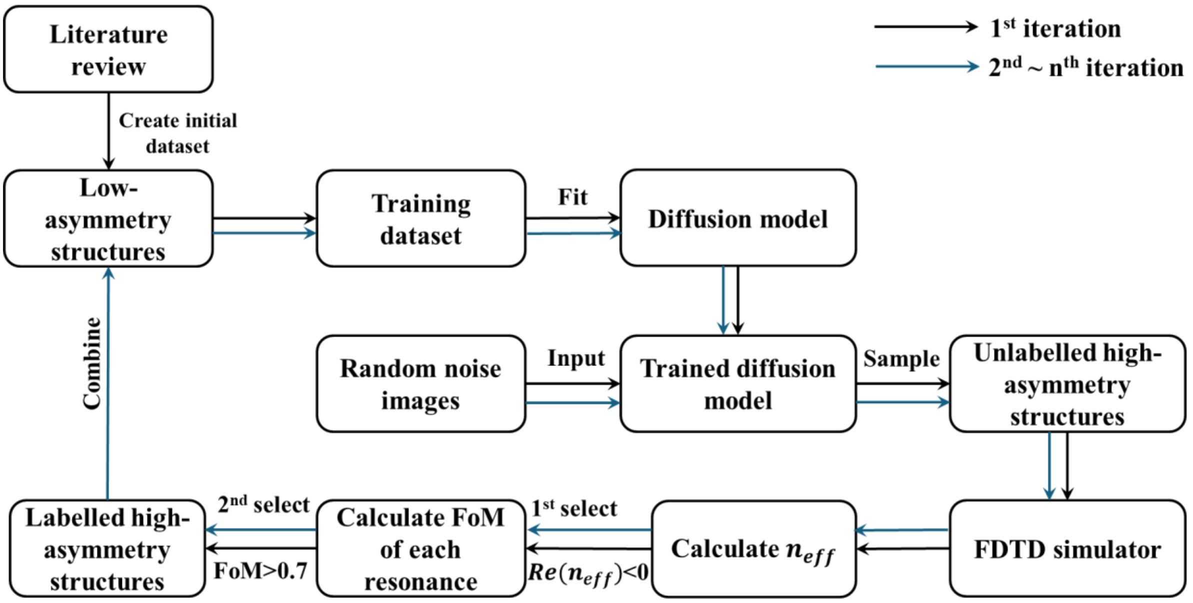

Figure S1. Flowchart of implementing the proposed active learning-augmented diffusion model for generating high-asymmetry structures with high-performance THz resonance.

\

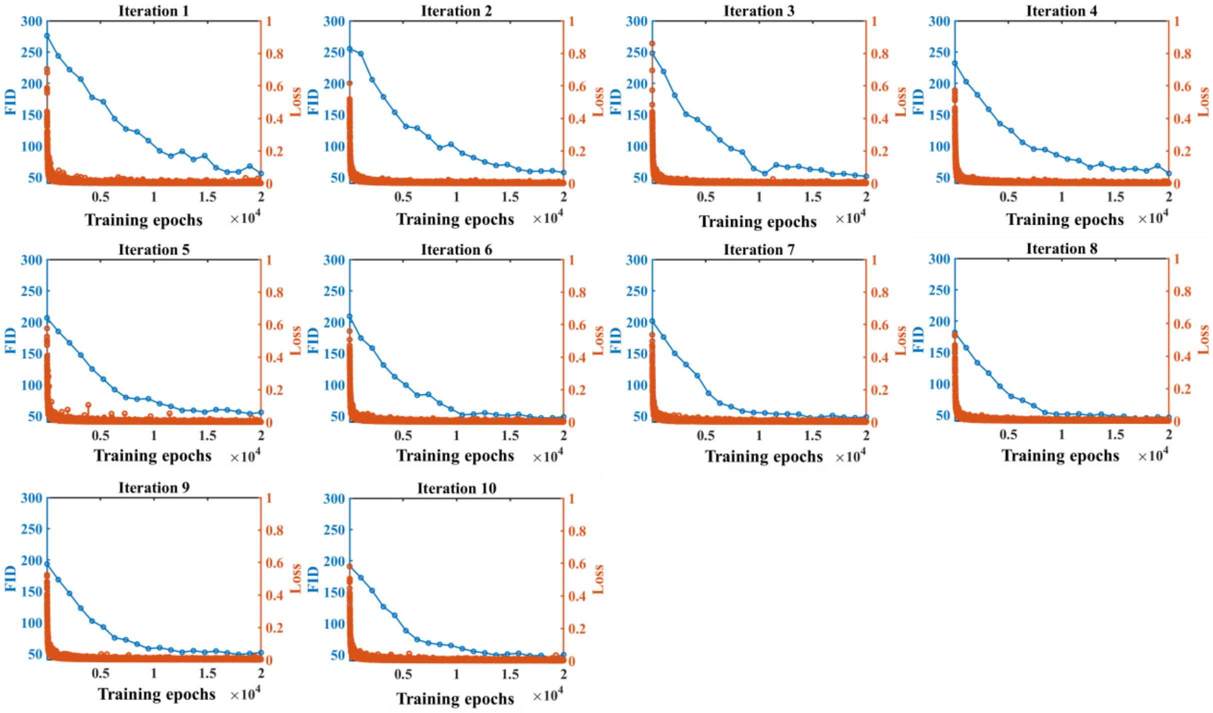

Figure S2. The curves of training loss and FID with training epochs in each iteration.

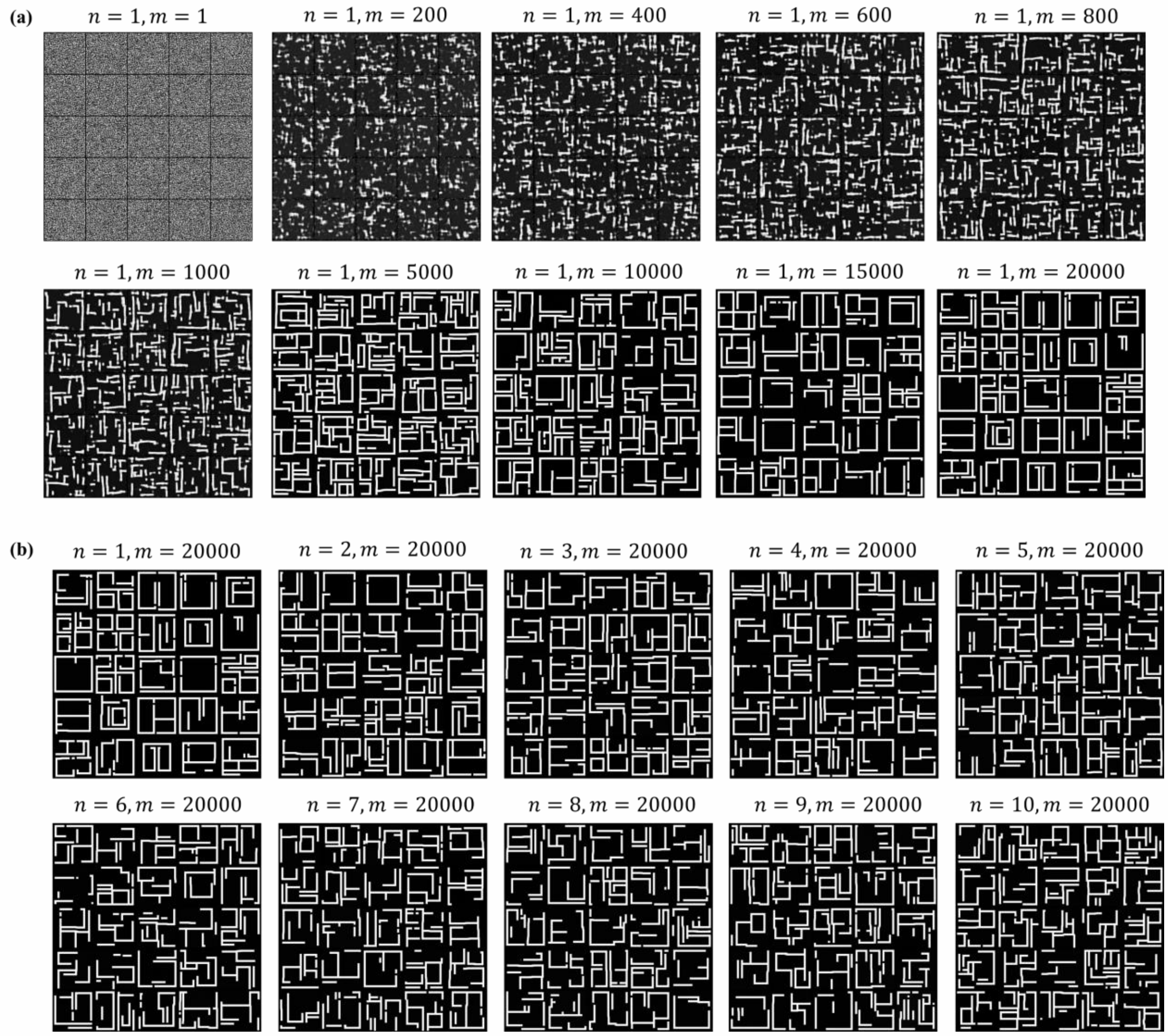

Figure S3. The visual results of generated samples with (a) increasing training epochs in a single iteration. (b) increasing iterations and at a specific training epoch with the lowest FID score.

Supplementary Note 3: Algorithms of Diffusion Model.

In the training algorithm of a diffusion model, the variational lower bound (VLB) is used to optimize the negative log-likelihood. We use $q(\mathbf{x}_t|\mathbf{x}_{t-1})$ to represent a forward step with a normal distribution with mean $\sqrt{1-\beta_t}\mathbf{x}_{t-1}$ and covariance $\beta_t\mathbf{I}$ generating the structural image $\mathbf{x}_t$ and $p_\theta(\mathbf{x}_{t-1}|\mathbf{x}_t)$ to represent a reverse step with a normal distribution with mean $\boldsymbol{\mu}_\theta(t,\mathbf{x}_t)$ and covariance $\boldsymbol{\Sigma}_\theta(t,\mathbf{x}_t)$ reconstructing the structural image $\mathbf{x}_{t-1}$. The loss function τ_{VLB} for estimating the reverse step can be expressed as[79-82]

$$\tau_{VLB} = \mathbb{E}_{q(\mathbf{x}_{0:T})}\left[\log\frac{q(\mathbf{x}_{1:T}|\mathbf{x}_0)}{p_\theta(\mathbf{x}_{0:T})}\right] \geq \mathbb{E}_{q(\mathbf{x}_0)}[-\log p_\theta(\mathbf{x}_0)], \tag{S3}$$

where $\mathbb{E}$ represents the expected value. Using the Kullback-Leibler (KL) divergence to rewrite Equation S3, the objective function can be derived as

$$\begin{aligned}\tau_{VLB} = \mathbb{D}_{KL}\big(q(\mathbf{x}_T|\mathbf{x}_0) \parallel p_\theta(\mathbf{x}_T)\big) + \sum_{t=2}^{T}\mathbb{E}_{q(\mathbf{x}_t|\mathbf{x}_0)}\big[\mathbb{D}_{KL}\big(q(\mathbf{x}_{t-1}|\mathbf{x}_t,\mathbf{x}_0) \parallel p_\theta(\mathbf{x}_{t-1}|\mathbf{x}_t)\big)\big] \\ + \mathbb{E}_{q(\mathbf{x}_1|\mathbf{x}_0)}[-\log p_\theta(\mathbf{x}_0|\mathbf{x}_1)] = \tau_T + \sum_{t=1}^{T-1}\tau_t + \tau_0,\end{aligned} \tag{S4}$$

where $\mathbb{D}_{KL}$ denotes the KL divergence. τ_T is a constant and hence can be ignored during the training process. When the covariance and the mean of each reverse step are set as

$$\boldsymbol{\Sigma}_\theta(t,\mathbf{x}_t) = \sigma_t^2\mathbf{I} = \beta_t\mathbf{I}, \tag{S5}$$

$$\boldsymbol{\mu}_\theta(t,\mathbf{x}_t) = \frac{1}{\sqrt{\alpha_t}}\left(\mathbf{x}_t - \frac{\beta_t}{1-\hat{\alpha}_t}\boldsymbol{\epsilon}_\theta(\mathbf{x}_t,t)\right), \tag{S6}$$

where $\boldsymbol{\epsilon}_\theta(\mathbf{x}_t,t)$ is the predicted noise by the network with the trainable parameters θ from an input image $\mathbf{x}_t$, the loss term τ_t that aims to minimize the distance between the real mean $\boldsymbol{\mu}_t(\mathbf{x}_0,\mathbf{x}_t)$ and the predicted mean $\boldsymbol{\mu}_\theta(t,\mathbf{x}_t)$ can be parameterized as

$$\tau_t = \mathbb{E}_{\mathbf{x}_0,\boldsymbol{\epsilon}_t}\left[\frac{1}{2\|\boldsymbol{\Sigma}_\theta(t,\mathbf{x}_t)\|_2^2}\|\boldsymbol{\mu}_t(\mathbf{x}_0,\mathbf{x}_t) - \boldsymbol{\mu}_\theta(t,\mathbf{x}_t)\|^2\right], \tag{S7}$$

where $\boldsymbol{\epsilon}_t$ is the real noise at time step t. Then after ignoring the weighting term, the objective can be simplified to[79-82]

$$\tau_t = \mathbb{E}_{t\in[1,T],\mathbf{x}_0,\boldsymbol{\epsilon}_t}\left[\left\|\boldsymbol{\epsilon}_t - \boldsymbol{\epsilon}_\theta\big(\sqrt{\hat{\alpha}_t}\mathbf{x}_0 + \sqrt{1-\hat{\alpha}_t}\boldsymbol{\epsilon}_t, t\big)\right\|^2\right], \tag{S8}$$

where $\alpha_t = 1-\beta_t$, $\hat{\alpha}_t = \prod_{i=1}^{t}\alpha_i$, $\boldsymbol{\epsilon}_t$ is the real noise at the time step t, and $\boldsymbol{\epsilon}_\theta$ is the predicted noise at time step t. Based on Equation S8, the detailed algorithms of training the diffusion

model and sampling high-asymmetry structures using the trained diffusion model are shown in **Figure S4**. Algorithm 1 illustrates the process of training the diffusion model with the classical THz metamaterial structures collected from existing studies for n iterations. Algorithm 2 presents the sampling process of high-asymmetry structures generated by the trained model from input random noise images in the n iterations.

Algorithm 1 Training Diffusion Model with Classical Structures

Input: Training dataset $q(\mathbf{x}_0)$
Output: Model parameters θ
for $Iteration = 1, \ldots, n$ **do**
    repeat
        $\mathbf{x}_0 \sim q(\mathbf{x}_0)$
        $t \sim \mathrm{Uniform}(\{1, \ldots, T\})$
        $\epsilon_t \sim \mathcal{N}(\mathbf{0}, \mathbf{I})$
        Take gradient descent step on
        $\nabla_\theta \| \epsilon_t - \epsilon_\theta(\sqrt{\hat{\alpha}_t}\mathbf{x}_0 + \sqrt{1-\hat{\alpha}_t}\epsilon_t, t)\|^2$
    until converged
end for

Algorithm 2 Sampling High-Asymmetry Structures Using the Trained Diffusion Model

Input: Random noise image $\mathbf{x}_T \sim \mathcal{N}(\mathbf{0}, \mathbf{I})$
Output: High-asymmetry structural image $\mathbf{x}_0$
for $Iteration = 1, \ldots, n$ **do**
    for $t = T, \ldots, 1$ **do**
        $\mathbf{z} \sim \mathcal{N}(\mathbf{0}, \mathbf{I})$ if $t > 1$, else $\mathbf{z} = \mathbf{0}$
        $\mathbf{x}_{t-1} = \frac{1}{\sqrt{\alpha_t}}\left(\mathbf{x}_t - \frac{1-\alpha_t}{\sqrt{1-\hat{\alpha}_t}}\epsilon_\theta(\mathbf{x}_t, t)\right) + \sqrt{1-\alpha_t}\mathbf{z}$
    end for
    return $\mathbf{x}_0$
end for

Figure S4. Algorithms of training the diffusion model with the classical structures (Algorithm 1) and then sampling the high-asymmetry structures using the trained diffusion model (Algorithm 2).

Supplementary Note 4: Network Architecture.

The architecture of the residual U-shaped network (U-Net) adopted for generating the structural image in each diffusion step is listed in **Table S1**. The dimension of the structural image that represents one unit cell of the metamaterial is 100×100 and each pixel represent 1×1 μm^2. A general U-Net consists of four encoding modules to extract the features of the input structural images and four decoding modules to reconstruct the output structural image. Each encoding module has two 3×3 convolutional layers followed by one 2×2 down-sampling layer, and each decoding module has one 2×2 up-sampling layer followed by two 3×3 convolutional layers. The residual U-Net involved in the diffusion model uses the Wide Residual Network (ResNet) with residual convolutions shown in Figure 3 of the manuscript to replace the general convolutional layers in each encoding or decoding module, which effectively increases the network depth while avoiding the gradient vanishing problem. The skip connections between the encoder and the decoder are designed to avoid information loss in the down-sampling process of the encoder, which is implemented by concatenating the feature maps at the same scale in the encoding and decoding module. In the reverse diffusion process, the residual U-Net is used to predict and remove the noise image step by step, and the structural image can be finally reconstructed.

Table S1. Network architecture of the residual U-Net adopted in the diffusion model.

Network	Modules	Layers	Input	Output	Kernel
Encoder	Encoding 1	Wide ResNet 1 [a]	1×100×100	64×100×100	64×3×3
		Wide ResNet 2	64×100×100	64×100×100	64×3×3
		Down-sampling 1	64×100×100	64×50×50	-
	Encoding 2	Wide ResNet 3	64×50×50	128×50×50	128×3×3
		Wide ResNet 4	128×50×50	128×50×50	128×3×3
		Down-sampling 2	128×50×50	128×25×25	-
	Encoding 3	Wide ResNet 5	128×25×25	256×25×25	256×3×3
		Wide ResNet 6	256×25×25	256×25×25	256×3×3
		Down-sampling 3	256×25×25	256×12×12	-
	Encoding 4	Wide ResNet 7	256×12×12	512×12×12	512×3×3
		Wide ResNet 8	512×12×12	512×12×12	512×3×3
		Down-sampling 4	512×12×12	512×6×6	-
		Conv 1 [b]	512×6×6	1024×6×6	1024×3×3
	Bridge	Conv 2	1024×6×6	1024×6×6	1024×3×3
Decoder	Decoding 1	Up-sampling 1	1024×6×6	512×12×12	-
		Concat 1 [c]	512×12×12	1024×12×12	-
		Wide ResNet 9	1024×12×12	512×12×12	512×3×3
		Wide ResNet 10	512×12×12	512×12×12	512×3×3
	Decoding 2	Up-sampling 2	512×12×12	256×25×25	-
		Concat 2	256×25×25	512×25×25	-
		Wide ResNet 11	512×25×25	256×25×25	256×3×3
		Wide ResNet 12	256×25×25	256×25×25	256×3×3
	Decoding 3	Up-sampling 3	256×25×25	128×50×50	-
		Concat 3	128×50×50	256×50×50	-
		Wide ResNet 13	256×50×50	128×50×50	128×3×3
		Wide ResNet 14	128×50×50	128×50×50	128×3×3
	Decoding 4	Up-sampling 4	128×50×50	64×100×100	-
		Concat 4	64×100×100	128×100×100	-
		Wide ResNet 15	128×100×100	64×100×100	64×3×3
		Wide ResNet 16	64×100×100	64×100×100	64×3×3
	Output	Conv 3	64×100×100	1×100×100	1×1×1

[a] The structure of Wide ResNet has been demonstrated in Figure 3. [b] "Conv" represents convolutional layer. [c] "Concat" means the concatenation of the feature maps at the same scale in the encoding and decoding modules.

Supplementary Note 5: Classical Structures in Initial Training Dataset.

Figure S5 presents the classical structures used in the initial training dataset and their corresponding transmissions, in which the metal resonators use 200 nm thick aluminum (Al) with the substrate silicon dioxide (SiO_2). We collect 14 types of metamaterial structures with THz resonance.[16-35] To enlarge the dataset, the geometric parameters of each structure are adjusted as shown in Figure S5. According to the simulation verification that ensures the resonance working in the frequency range of [0.3, 0.8] THz, we adopt 6, 4, 16, 3, 3, 2, 4, 11, 4, 6, 5, 2, 1, and 1 structural image with various geometric parameters of the 1st to 14th types of structures, respectively. Therefore, there are in total 68 structural images used for the 1st iteration of training.

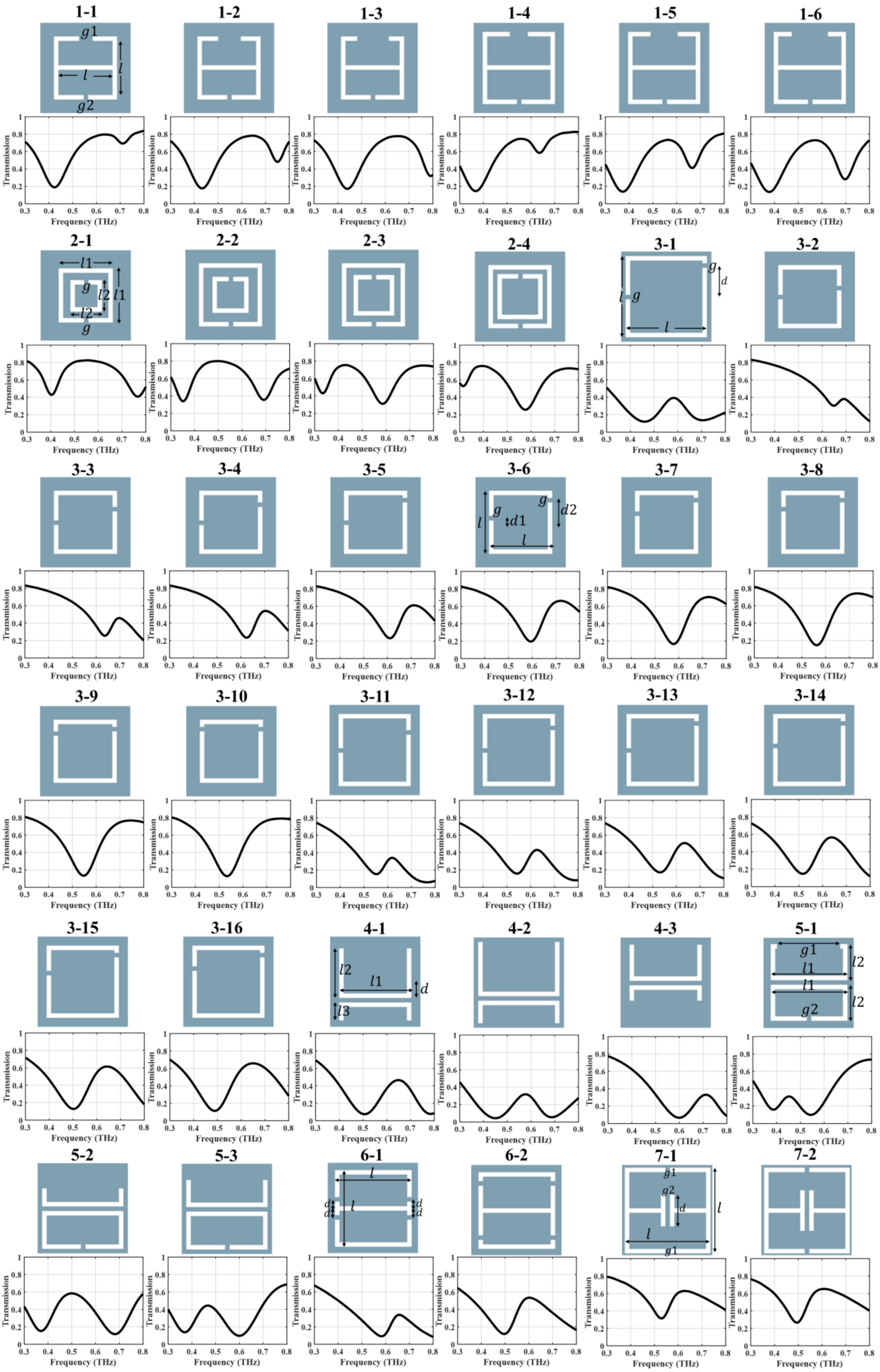
1-1
1-2
1-3
1-4
1-5
1-6
2-1
2-2
2-3
2-4
3-1
3-2
3-3
3-4
3-5
3-6
3-7
3-8
3-9
3-10
3-11
3-12
3-13
3-14
3-15
3-16
4-1
4-2
4-3
5-1
5-2
5-3
6-1
6-2
7-1
7-2
Transmission
Frequency (THz)

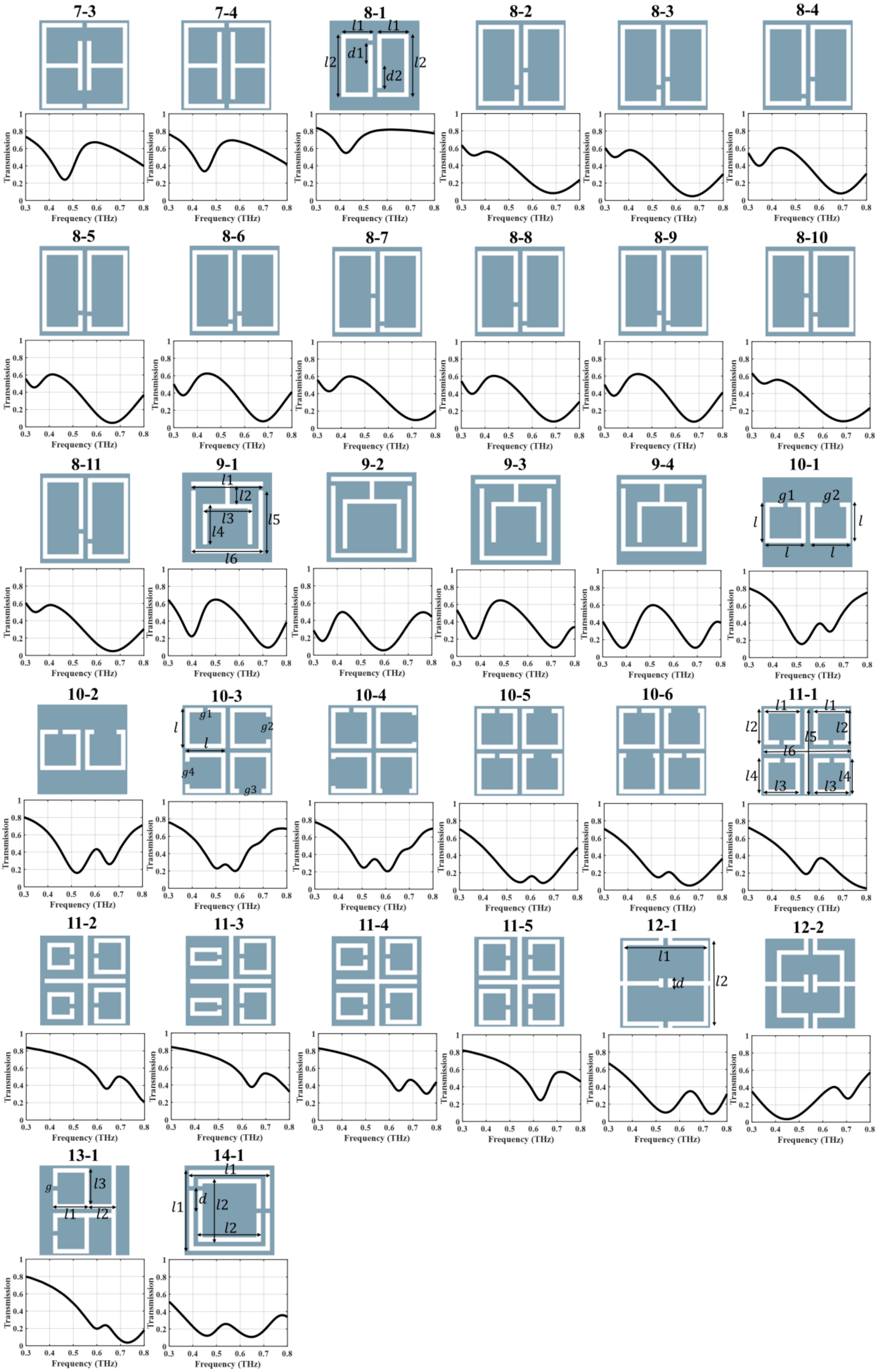
7-3
7-4
8-1
8-2
8-3
8-4
8-5
8-6
8-7
8-8
8-9
8-10
8-11
9-1
9-2
9-3
9-4
10-1
10-2
10-3
10-4
10-5
10-6
11-1
11-2
11-3
11-4
11-5
12-1
12-2
13-1
14-1
Transmission
Frequency (THz)

Figure S5. 14 types of classical metamaterial structures and their simulated transmissions. A total of 68 structural images with different geometrical parameters are used in the initial training dataset.

To present the typical characteristics of these classical resonances, we conducted the simulations using PEC and Al for classical structures involved in the training dataset and three examples are demonstrated in **Figure S6**. First, the BIC and Fano resonance realized by the SRR structures with double splits proposed in [21-23] are indicated in Figure S6a. The non-zero $d1$ breaks the symmetry of the structure and introduces asymmetric profile typical of Fano resonances, especially obvious in the simulations with PEC without material loss. Besides, the extremely narrow and high-Q resonance of QBIC can be observed when $d1 = 5$, and the Q factor decreases as $d1$ increases. Second, the sharp resonances associated with toroidal dipoles realized by the structure proposed in [34] are demonstrated in Figure S6b. We can observe the resonance tends to disappear when increasing d from 0 to 2 and then re-appear when increasing d from 2 to 12, which is consistent with the analysis in [34]. Third, the transparent window featuring characteristic of EIT is presented in Figure S6c, caused by the coupling of two sub-resonators as proposed in [31]. In this work, we have adjusted the parameters of each classical structure in the simulation with Al to ensure the resonance in the frequency range of 0.3-0.8 THz and selected 68 structures with high-FoM resonance as shown in Figure S5 as the initial training dataset.

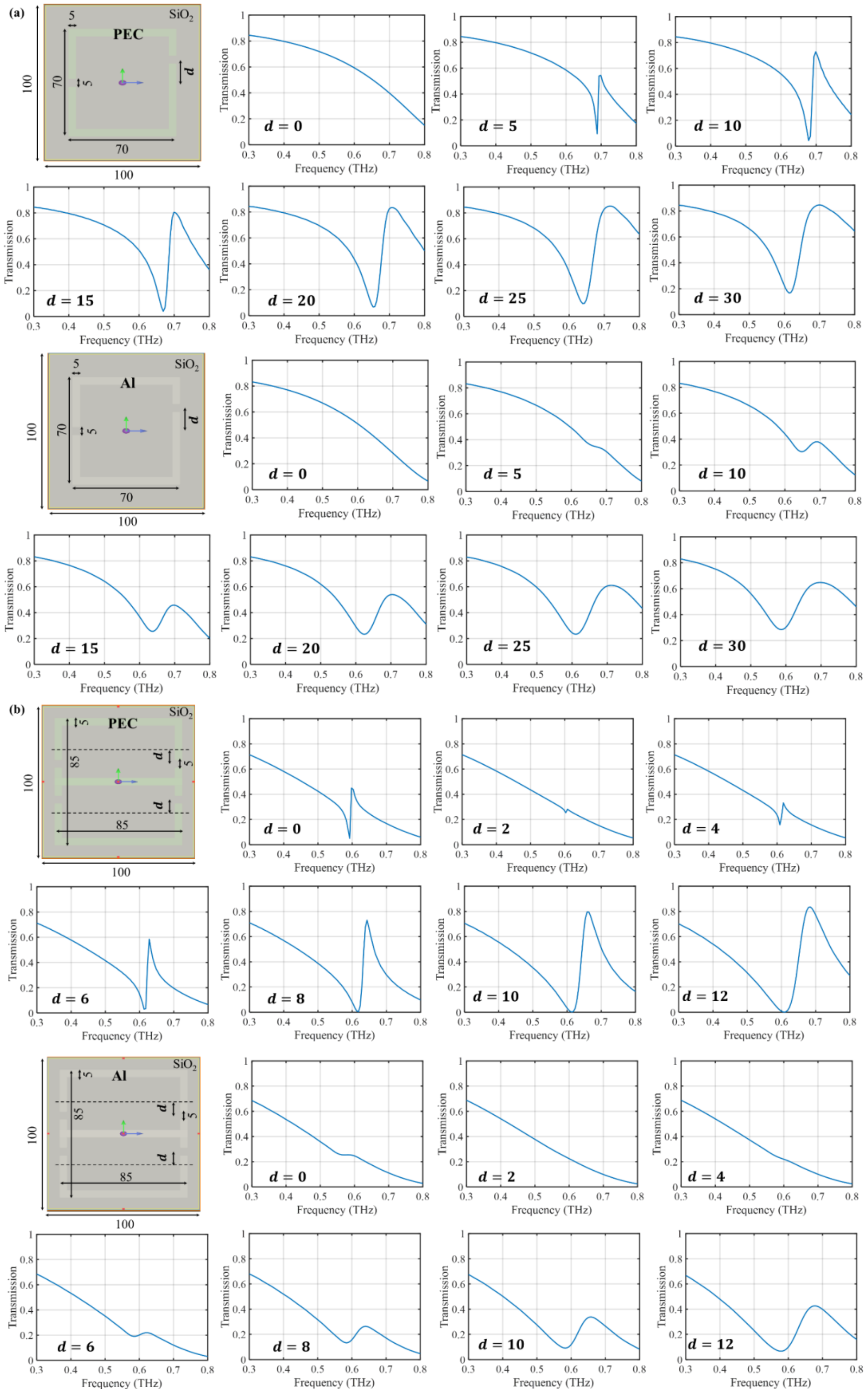
(a)
SiO2
PEC
Al
5
70
100
d
Transmission
Frequency (THz)
d = 0
d = 5
d = 10
d = 15
d = 20
d = 25
d = 30
(b)
85
d = 0
d = 2
d = 4
d = 6
d = 8
d = 10
d = 12

(c)

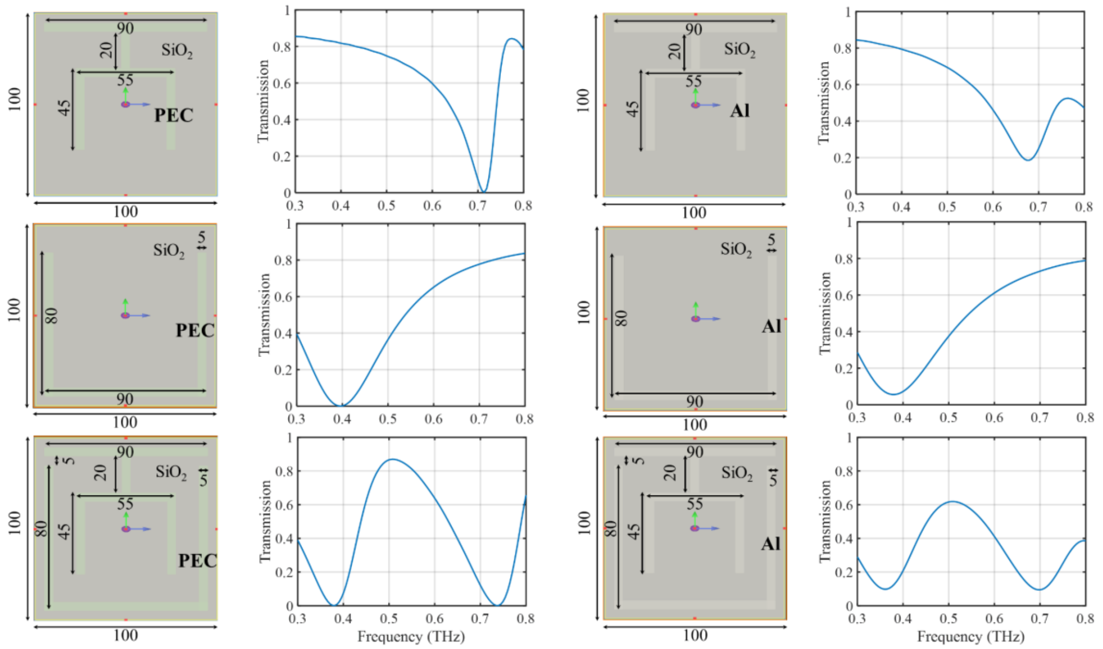

Figure S6. Simulation results for classical structures with typical resonance characteristics using PEC and Al as the resonator materials, including (a) BIC and Fano resonances, (b) toroidal dipoles, and (c) EIT.

Supplementary Note 6: More Trip-Resonance Structures.

More newly generated high-asymmetry structures with triple resonance and the corresponding transmissions are shown in **Figure S7**, and we have calculated the resonance metrics for each resonance. As previously shown in Figure S5, the structures in the initial training dataset only involve single or double resonances. Due to the powerful learning capability of the generative model, new triple-resonance structures can be produced using the trained diffusion model. However, as the generative network has never seen tripe-resonance structures in the training process, its generative capability for tripe-resonance structures is limited. In our case, nearly only one structure in one hundred samples can have tripe resonances. Besides, as shown in Figure S7, the overall Q, FoM, and improved figure of merit (IFoM) of triple-resonance structures are relatively lower than those of single- or double-resonance structures. These limitations are expected, given that the generative model has not been exposed to such resonance characteristics during training. However, the emergence of new triple-resonance structures is still highly significant, which shows the potential that we can use the proposed method to automatically achieve complex multiple resonance only requiring simple one- and two-resonance structures to form a small training set for the generative model. Adding classical structures with triple resonances to the initial training dataset can have the potential to solve these issues, enabling the generative network to learn features of triple-resonance structures and then generate new structures with triple resonances in higher performance.

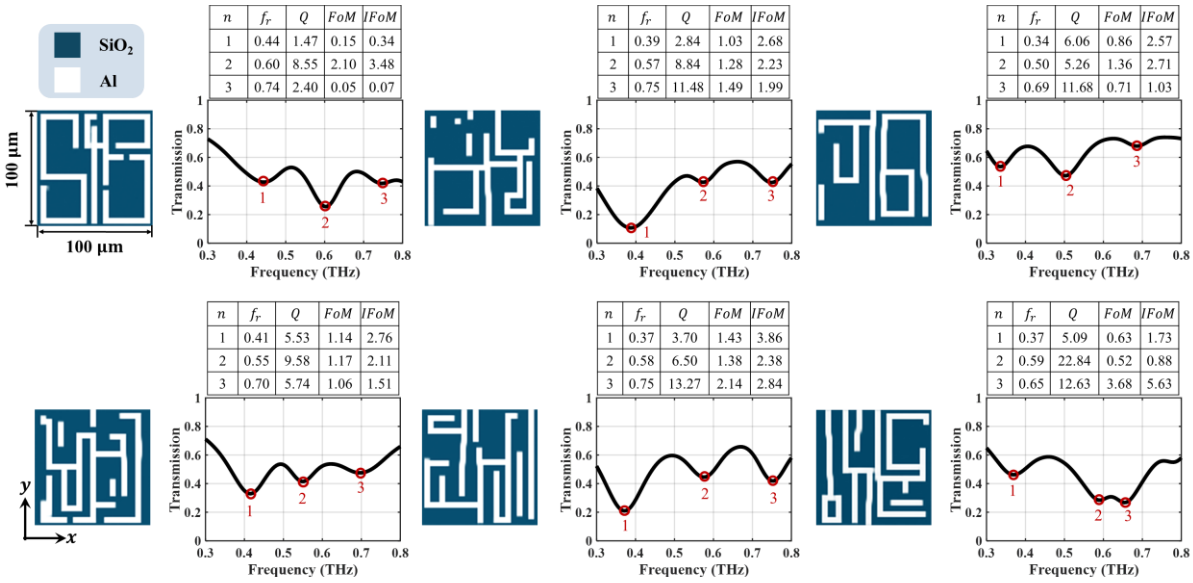

Figure S7. More generated high-asymmetry structures with trip resonances that never appear in the initial training dataset.

Furthermore, we would like to clarify that the occasional appearance of triple-resonance structures is not by coincidence but a meaningful outcome. Since no such structures were included in the training dataset, their emergence shows that our framework can go beyond what it has seen before. This indicates that even when trained only on simple cases (e.g., single- and double-resonance structures), the model can still generate more complex and hard-to-obtain designs. In practice, this means that if we start from easily available structures, our method can help uncover advanced candidates that would otherwise be very difficult to design manually. Besides, although the frequency of triple-resonance occurrence is low, it is also stable. To verify this, we trained the model for ten more iterations, in which 100 structures are generated in each iteration, and recorded the results, as shown in **Table S2**. The table shows that the occurrence rate fluctuates with an average of about 1.3%. This consistency demonstrates that the phenomenon is reproducible, even though it happens rarely.

Table S2. Low but stable rate of triple-resonance occurrence in the active learning process.

Iteration	11	12	13	14	15	16	17	18	19	20	Ave.
Rate	1/100	2/100	1/100	0/100	1/100	0/100	1/100	3/100	3/100	1/100	1.3%

Supplementary Note 7: Microfabrication Process and Results.

Figure S8 illustrates the microfabrication process of each sample and **Figure S9** presents the microscopic images of all the fabricated samples. In total, we fabricated sixteen samples to demonstrate the THz resonance performance, including eight symmetric or low-asymmetry structures selected from the initial training dataset as shown in Figure S9a and eight high-asymmetry structures produced by the generative model as shown in Figure S9b. It should be noted that the fabrication costs of the classical structures and the generated complex structures are the same.

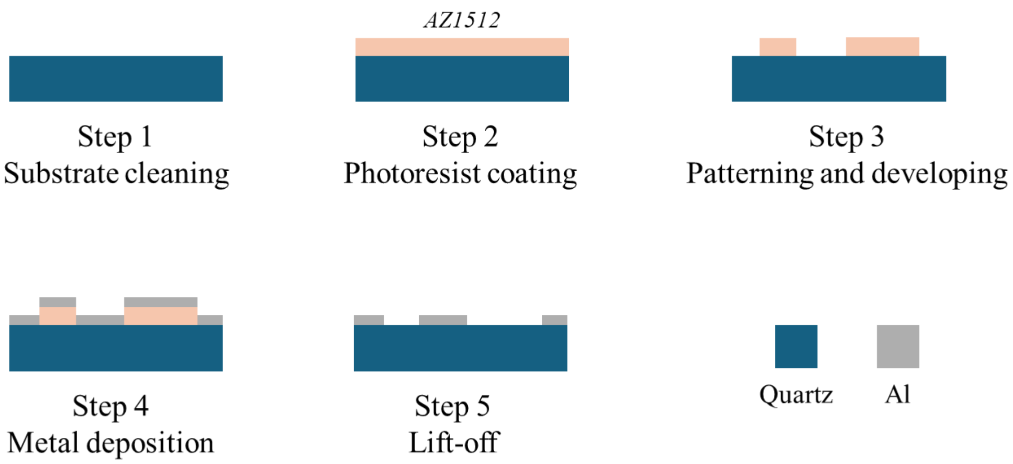

Figure S8. Flowchart of the sample fabrication process.

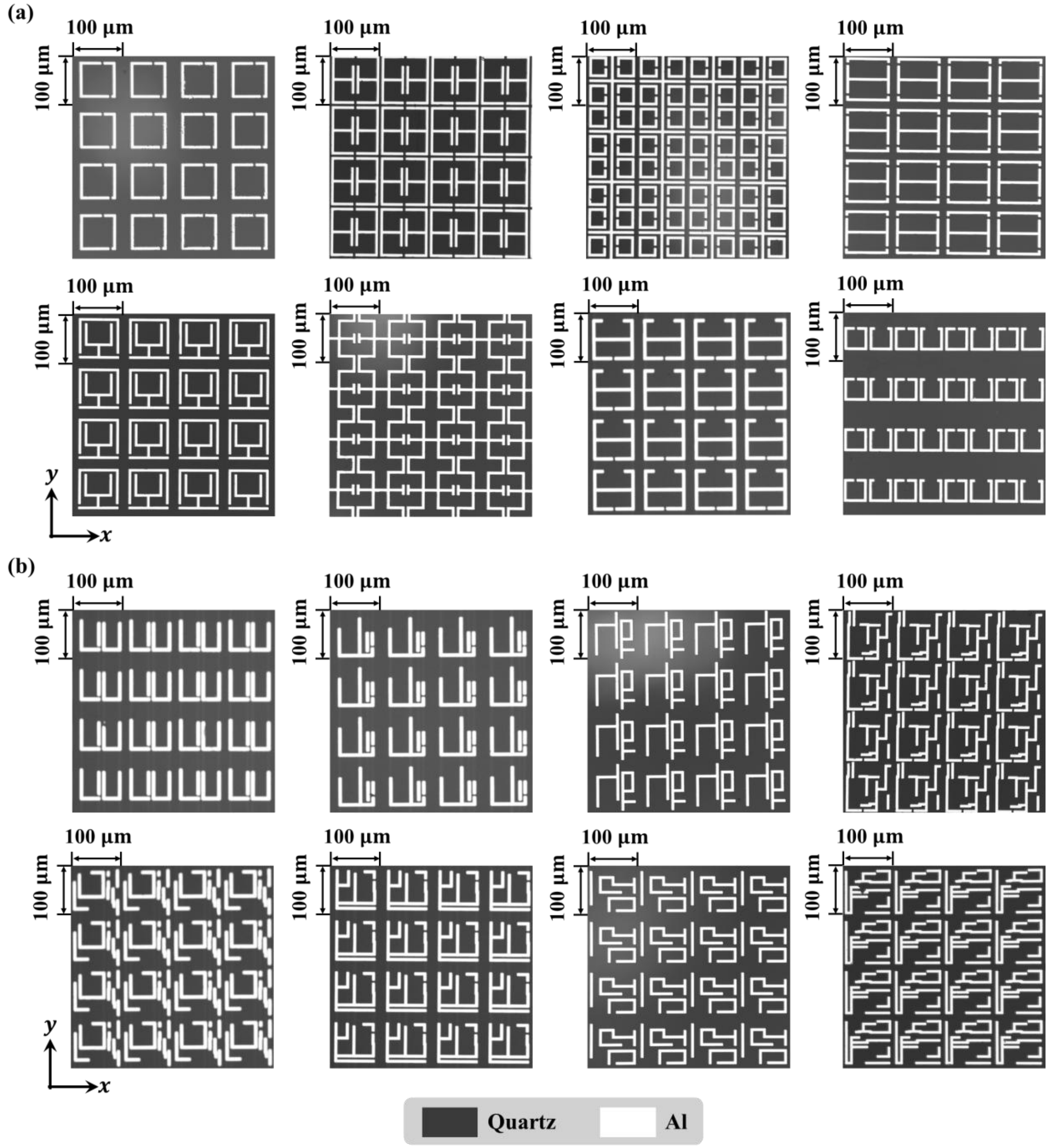

Figure S9. Microscopic images of the sixteen fabricated samples including a) the traditional symmetric or low-asymmetry structures and b) the generated high-asymmetry structures.

Supplementary Note 8: THz Measurement Setup and Original Measurement Data

The commercial photoconductive antenna-based THz spectroscopy setup for the experimental demonstration in this study is illustrated in **Figure S10**. We measured the co-polarized transmissions of the sixteen classical and high-asymmetry metamaterial samples as well as the bare quartz substrate. Each chip was mounted on the carrier with a circular aperture. The incoming x-polarized THz wave was incident normally on the sample and the transmitted x-polarized signal was detected. The original, unnormalized transmission spectra are provided in **Figure S11**, including those of the eight classical structures in Figure S11a and the eight new generated structures in Figure S11b. To fairly compare their resonance metrics, the transmission spectrum of each sample was normalized against that of the bare quartz substrate in the manuscript.

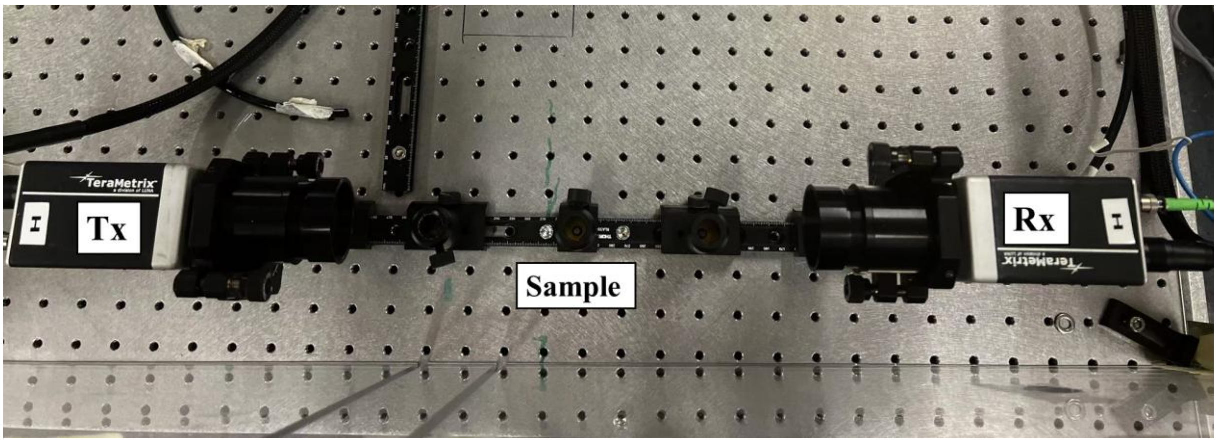

Figure S10. Experimental setup for the THz measurement of co-polarized transmission.

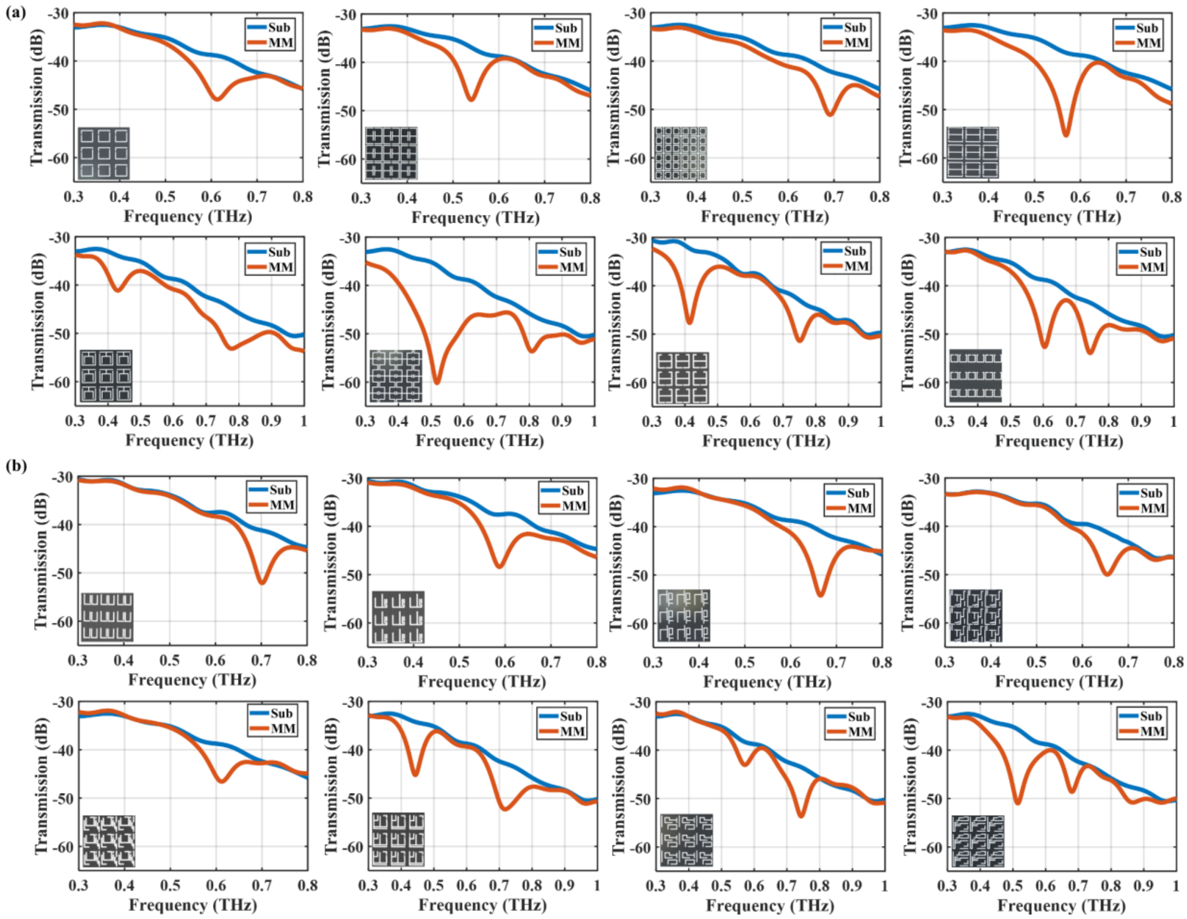

Figure S11. The original experimental transmission spectra without normalizations of (a) the eight classical structures and (b) the eight generated structures as well as the bare quartz substrate without any metamaterial structures. “Sub” and “MM” represents the quartz substrate and the metamaterial structures, respectively.

Supplementary Note 9: Investigations on Potential Sensing Applications.

To demonstrate the potential of the proposed scheme on practical sensing applications, we further examine the sensitivity of the generated high-asymmetry structure. We simulate its transmissive response to varying thicknesses of α-lactose coated on its surface and the results are shown in **Figure S12**. The complex permittivity (real and imaginary components) of α-lactose in the 0.3–0.8 THz range is shown in Figures S12a and S12b.[83] Notably, α-lactose exhibits a characteristic resonance near 0.53 THz. Using this material model, we performed FDTD simulations by coating the metamaterial surface with α-lactose layers of thicknesses ranging from 2 µm to 20 µm with a step of 2 µm. The resulting transmission spectra are shown in Figure S12c, while the corresponding resonance frequency shifts are plotted in Figure S12e. The resonance frequency shift increases rapidly at first and then gradually saturates. To further analyze the resonance peak variation of α-lactose itself, we repeated the simulations with the imaginary part of the permittivity set to zero over the same frequency range, effectively suppressing absorption. The resulting spectra are normalized against the original ones from Figure S12c, then we can observe the resonance peaks of various thicknesses of α-lactose in Figure S12d. The resonance peaks at 0.53 THz with the α-lactose thickness are plotted in Figure S12f, showing a sharp decrease in amplitude with increasing thickness, followed by a steady-state behavior. These results demonstrate the potential of the generated high-asymmetry structure to detect common analytes like α-lactose that exhibit resonant absorption features within the 0.3–0.8 THz spectral range.

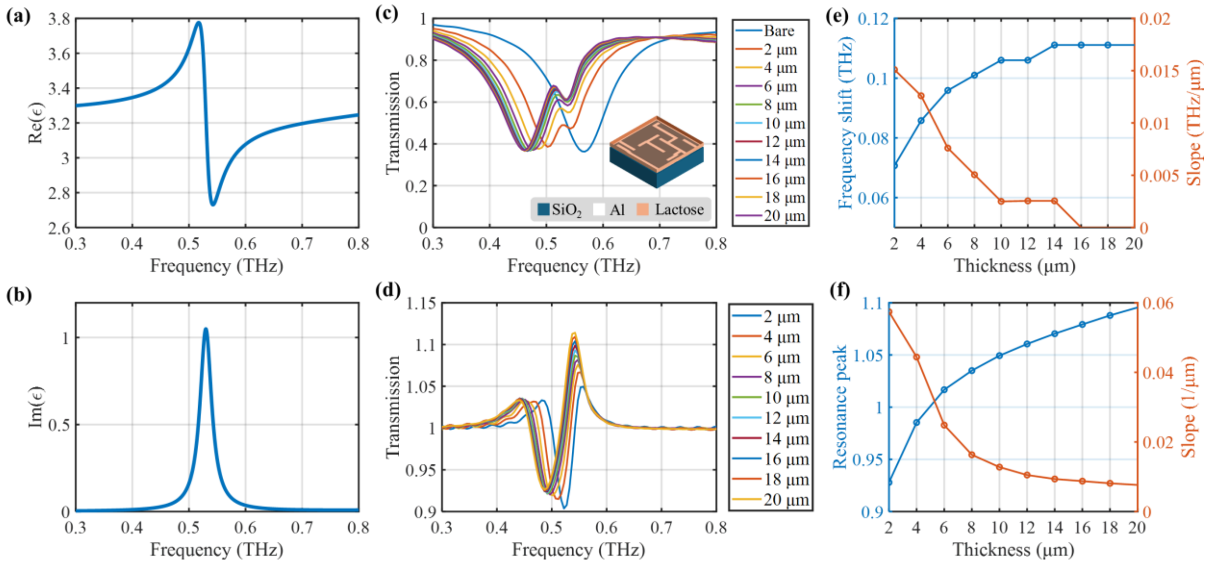

Figure S12. The simulation results of coating α-lactose with various thicknesses on the surface of the generated high-asymmetry metamaterial structure. (a) The real part and (b) the imaginary part of the

permittivity (ϵ) of α-lactose in the frequency range of 0.3-0.8 THz. (c) The transmission spectra when various thicknesses of α-lactose are coated on the surface. (d) The transmission spectra normalized against those of α-lactose under the setting of $\mathrm{Im}(\epsilon) = 0$. (e) The resonance frequency shift of the metamaterial structure with the thickness of α-lactose according to (c) and their slopes. (f) The resonance peaks of α-lactose with the coated thickness according to (d) and their slopes.

Supplementary Note 10: Comparative Study of Generative Models.

To clarify why we use the diffusion model rather than VAEs or GANs, we have conducted a comparative study on the generative performance and computation costs of the classical VAE,[84]-[85] GAN,[86]-[87] and the diffusion model involved in this work. We use the initial 68 classical structures to train the three models until they reach convergence, respectively. The training loss curves with good convergence and the generated structural images using the well-trained VAE, GAN, and diffusion model are visualized in **Figures S13a-c**, respectively. It can be observed that the visual quality of the structural images generated by the diffusion model is better than VAE and GAN, which is very important for metamaterial design as the binary images with high clarity and little noise will be easily imported in the subsequent simulation and fabrication. Furthermore, we compare the computational costs and evaluation metrics of these three models and show the results in **Table S3**. The FID measures distributional distance between the training dataset and the generated images by comparing their deep features extracted using a pretrained Inception-V3 network, while Learned Perceptual Image Patch Similarity (LPIPS) measures the perceptual similarity between random pairs of the generated images by comparing their deep features from a pretrained AlexNet network, weighted to match human visual perception.[88] Therefore, a lower FID represents better realism and quality of the generated images, and a higher LPIPS score represents better fidelity and intra-set diversity of the generated images. We can observe that the diffusion model achieves the lowest FID and highest LPIPS among the three models. As for the computational costs, the computational time of sampling each image using the diffusion model (0.4274 s) is indeed much higher than those using the VAE (0.0221 s) and GAN (0.0216 s). However, the generation for each structure with diffusion is still fast (below one second), which is acceptable for realizing rapid design of metamaterial design.

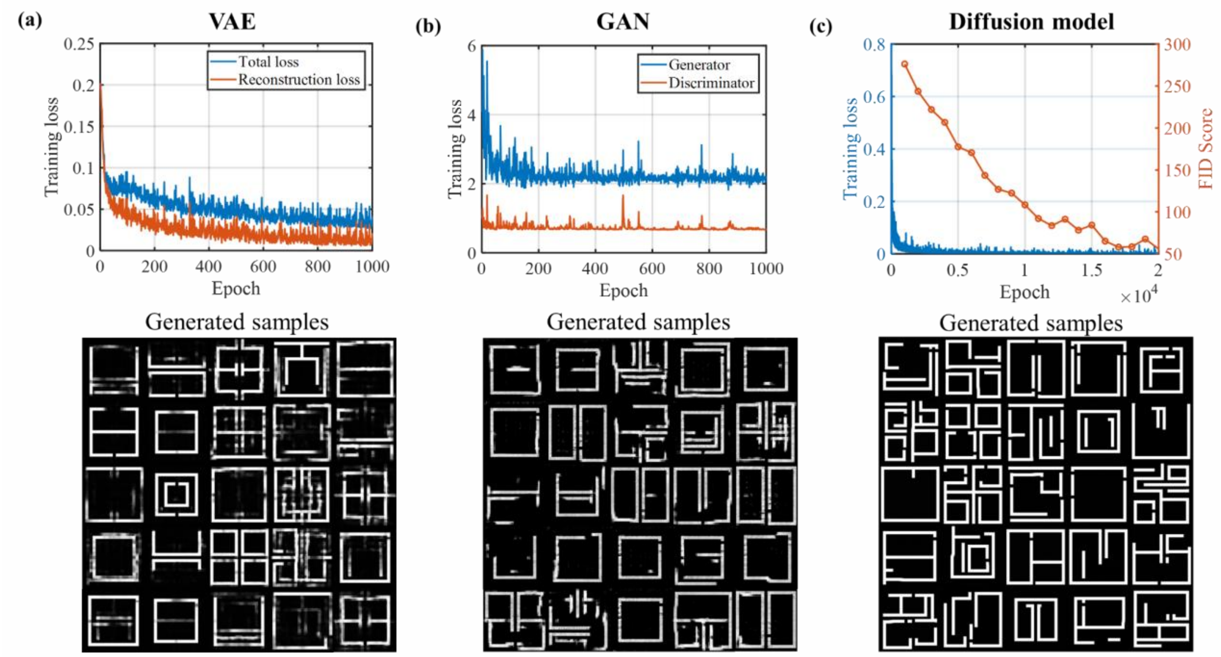

Figure S13. Comparative study for VAE, GAN and the diffusion model. (a) The training loss curves of VAE and the generated samples using the well-trained VAE model. (b) The training loss curves of GAN and the generated samples using the well-trained GAN model. (c) The training loss and FID score curves of the diffusion model and the generated samples using the well-trained diffusion model.

Table S3. Metrics and computational cost comparison for the generative performance of VAE, GAN, and the diffusion model.

**Model**	**Trainable Parameters**	**Training Time (s)**	**Sampling Time (s)**	**FID (↓)**	**LPIPS (↑)**
VAE	182,737,889	3666.54	0.0221	177.61	0.5760
GAN	7,962,240	5909.40	0.0216	225.37	0.5388
**Diffusion**	9,902,081	3588.42	0.4274	**55.21**	**0.5876**

Supplementary Note 11: Applicability of the Proposed Scheme to Different Target

To demonstrate its applicability to other structural design scenarios, we conduct an additional experiment for achieving resonance with high Q factors at a specific 0.5 THz and the comparative results are shown in **Figure S14**. We select 6 classical structures with resonance at 0.5 THz from the original 68 classical structures as the initial training dataset in this case. The proposed diffusion model is trained for 20,000 epochs in each iteration and 10 iterations in total. In each iteration of active learning, the well-trained diffusion model generates 10 samples, and we set the selection criterion for the second-step annotation to structures exhibiting a Q factor above 2.0. Other parameters remain unchanged. The curves of training loss and FID score with training epochs in each iteration are shown in Figure S14a, demonstrating good convergence. The curves of asymmetry and mean Q across iterations are shown in Figure S14b, in which Iteration 0 represents the initial classical structures. It can be observed the asymmetry of the generated structures increases and gradually reaches converged as the iteration rises. As for the mean Q, it reaches the maximum value in the fourth iteration, and the sharp jitter of the curve after the fourth iteration is due to too little data involved for the training. Figure S14c presents the comparison of mean and maximum Q values between the initial classical structures and the generated structures. Both the mean and maximum Q of the generated structures are higher than those of the initial dataset, reaching 6.50 and 10.59, respectively. Figures S14d-e demonstrate the transmission spectra of the 6 classical structures and 6 examples of the generated structures, respectively, with their Q factors indicated. These results demonstrate that the applicability of proposed model to different structural design scenarios, such as the high Q factor at a specific 0.5 THz. Once we constrain the training dataset within the desired target, the active learning-augmented diffusion model can learn the features of the provided training dataset and generate new structures with higher targeted metrics, caused by the generalization capability of the advanced generative model.

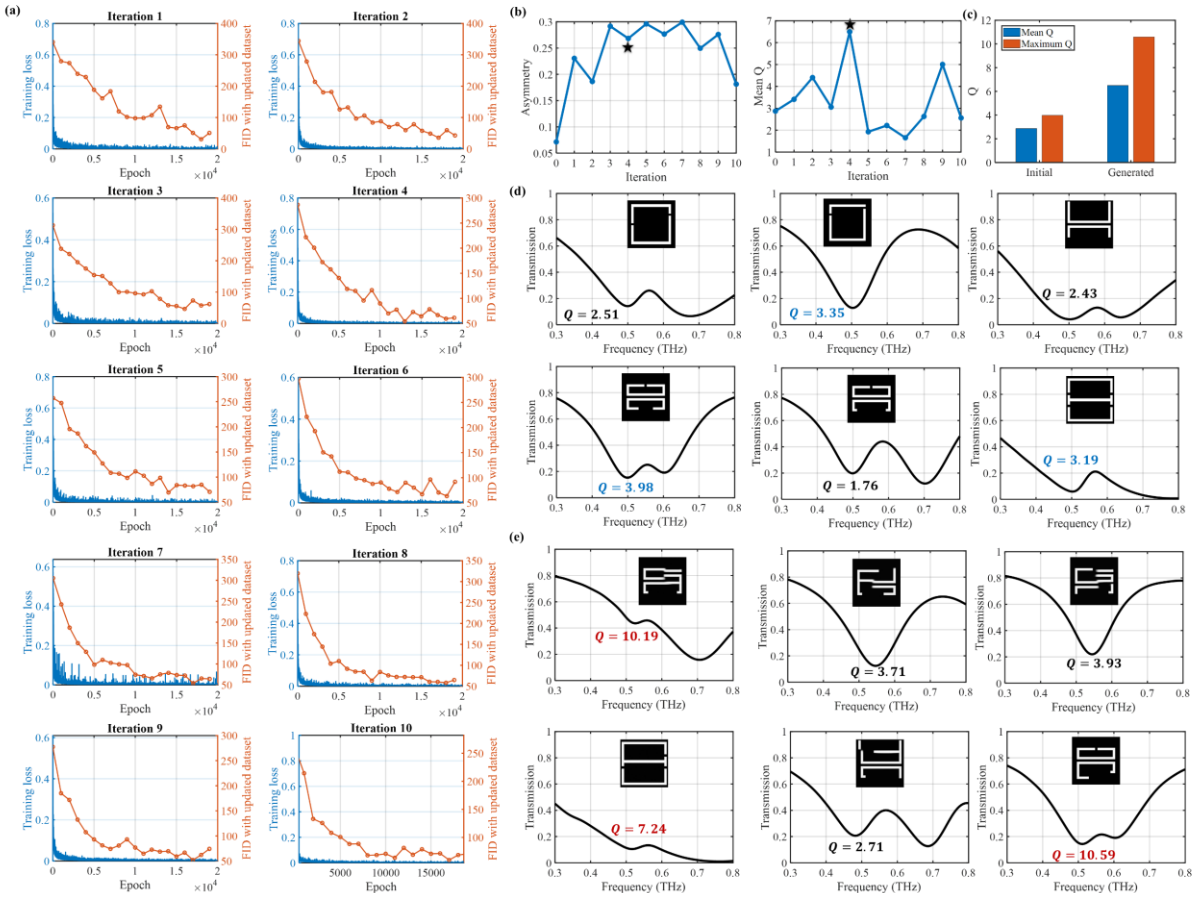

Figure S14. Comparative results when applying the proposed scheme to design new structure for achieving resonance at a specific 0.5 THz with high Q factors. (a) The training loss curves and FID score curves with the training epochs in the 10 iterations. (b) The asymmetry and mean Q curves across the active learning iterations. (c) Comparison of the mean and maximum Q factor between the initial structures and the generated structures. (d) The classical structures adopted in the initial training dataset and their transmissive spectrums with Q factors. (e) Selected high-asymmetry structures generated via the proposed model and their transmissive spectrums with Q factors.

Supplementary Note 12: Physical Analysis on Classical Structures

To confirm the claimed coupling phenomena of classical structures adopted in our initial training dataset, we have conducted physical analysis on representative structures corresponding to the classical resonance types (Fano, BIC, EIT, and toroidal dipole) as follows.

1) Fano resonance

To validate the physical origin of Fano resonance excited by Structure 3 [21-23], we perform spectral fitting and near-field simulations and present the results in **Figure S15**. To characterize the resonance observed in the transmission spectrum, both Fano and Lorentz models are fitted to the frequency-dependent transmittance within the range of 0.57–0.69 THz, as shown in Figure S15(a). The Fano model follows the canonical form with a linear background:

$$T(f) = A \cdot \frac{\left(q + \frac{2(f - f_0)}{\gamma}\right)^2}{1 + \left(\frac{2(f - f_0)}{\gamma}\right)^2} + C_0 + C_1 f \qquad (S9)$$

where A is the amplitude, f_0 is the resonance frequency, γ is the linewidth, q is the Fano asymmetry parameter, and $C_0 + C_1 f$ models the background. In contrast, the Lorentzian model is symmetric and lacks the interference term. The results show that the Fano model provides a significantly better fit (RMSE = 1.163×10^{-3}, $R^2 = 1.000$) compared to the Lorentzian model (RMSE = 5.325×10^{-3}, $R^2 = 0.994$), with a distinct asymmetric line shape around $f_0 = 0.659$ THz. The extracted quality factor is $Q = f_0/\gamma = 9.0$. The substantial differences in AIC and BIC values further validate that the resonance follows a Fano-type behavior, rather than a simple Lorentzian profile. Therefore, the spectral signature strongly supports the presence of a Fano resonance.

We further examined the near-field distributions of the transverse electric field E_y and surface current density K at frequencies near the resonance, as shown in Figure S15(b). The magnitude and phase maps of reveal distinct symmetry-breaking near 0.659 THz, including localized field enhancements and abrupt phase discontinuities. The anti-symmetric fraction η, computed from both E_y and K, shows a sharp drop in η_E from 0.55 to 0.18 at the resonance, while η_K remains nearly zero:

$$\eta = \frac{S_{\text{anti}}}{S_{\text{sym}} + S_{\text{anti}}} \qquad (S10)$$

Here, S_{sym} and S_{anti} denote the symmetric and anti-symmetric energy components, respectively. This indicates that the excitation source (current) remains symmetric, while the

electric field undergoes a radiation suppression due to destructive interference. Furthermore, the left–right phase difference $\Delta\phi/\pi$ for E_y undergoes a sharp phase jump across the resonance, which is another hallmark of Fano interference. Meanwhile, the phase of K remains constant, reinforcing that the observed asymmetry is not due to source excitation, but from modal interference. These near-field features, including the abrupt changes in field symmetry, phase flipping, and the decoupling between excitation and radiation, are all consistent with the interference mechanism that defines a Fano resonance. Therefore, Figures S15(a) and R1(b) together demonstrate that the resonance is Fano-type, arising from interference between a narrow discrete mode and a broad continuum.

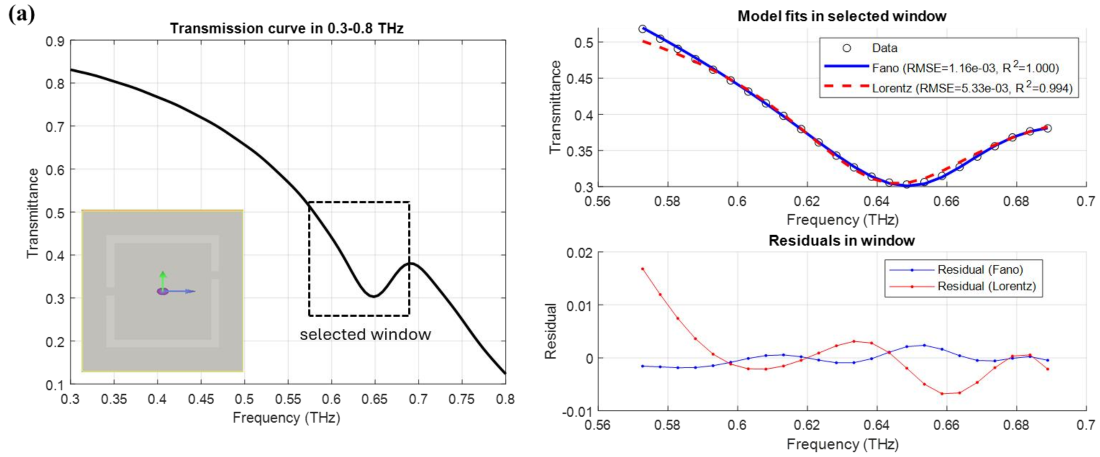

Model Fitting Comparison

Type	f_0	γ	q	Q	$RMSE$	R^2	AIC	BIC
Fano	0.659 THz	0.073 THz	0.438	9.0	1.164e-3	1.000	-312.30	-305.2
Lorentz	0.643 THz	0.074 THz	-	8.7	5.325e-3	0.994	-239.29	-232.2

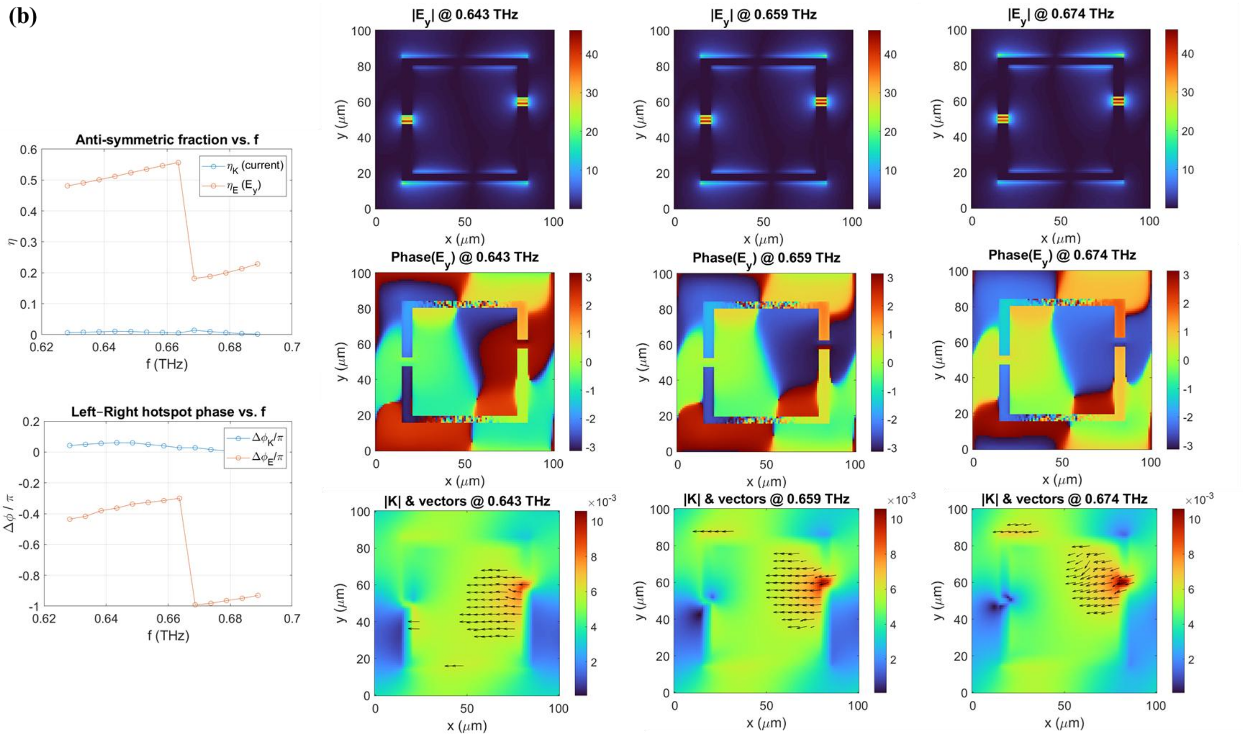

Figure S15. Physical analyses of Fano resonance. (a) The comparison of Fano and Lorentz resonance model fitting. (b) The near-field response analysis, including the transverse electric field E_y and surface current density K at frequencies near the resonance.

2) BIC

To further analyse the BIC phenomenon of Structure 3, we perform near-field simulation, energy flow analysis, and Q factor calculation with varying geometric parameter d of the structure. The results are shown in **Figure S16**. Figure S16(a) shows the electric field, magnetic

field, and Poynting vector when the structure is symmetric ($d = 0$ μm) with PEC material. Figure S16(b) shows the same analysis when the structure becomes slightly asymmetric ($d = 2$ μm). By comparing Figures S16(a) and R2(b), we can clearly observe the transition from a perfect BIC to a quasi-BIC. In the symmetric case ($d = 0$ μm), the fields are highly confined, and no energy escapes from the structure, indicating a non-radiative bound state. However, when a small asymmetry is introduced ($d = 2$ μm), the field distribution becomes imbalanced, and a noticeable energy flow appears at the edges. This energy leakage marks the emergence of a quasi-BIC, where the previously bound mode now weakly couples to the radiation continuum and starts to lose energy. This observation is also supported by the power leakage results: when $d = 0$ μm, the estimated leakage is about 5.33×10^{-8} (almost zero), while for $d = 2$ μm, it rises to 4.46×10^{-2}, indicating clear radiation loss. The total power leakage is calculated using the Poynting vector flux across the boundaries:

$$\Phi_{total} = \sum_{i=1}^{N_x} S_y(x_i, y_{min}) \cdot \Delta x + \sum_{i=1}^{N_x} S_y(x_i, y_{max}) \cdot \Delta x + \sum_{j=1}^{N_y} S_x\left(x_{min}, y_j\right) \cdot \Delta y + \sum_{j=1}^{N_y} S_x\left(x_{max}, y_j\right) \cdot \Delta y \quad (S11)$$

where S_x and S_y are the components of the time-averaged Poynting vector, Δx and Δy are the grid spacing, N_x and N_y are the number of grid points in x and y, and the four terms correspond to fluxes through the top, bottom, left, and right boundaries respectively.

Furthermore, Figures S16(c) and S16(d) present the Q factors with varying geometric parameter d that indicates the asymmetry of the structure with PEC and Aluminium (Al), respectively. In Figure S16(c), where the metal is set as a PEC, the Q factors are very high (over 100), especially when the structure is nearly symmetric. This agrees well with the expected behaviour of a BIC, which has very low energy loss and a very high Q. In contrast, Figure S16(d) shows the results when the metal is with electrical loss. In this case, the Q factors are much lower. Although a quasi-BIC still exists at small asymmetry, the Q is limited by material absorption. This comparison also explains why the Q factors of the classical structures used in our initial dataset are not very high, while they indeed exhibited the claimed classical coupling effects.

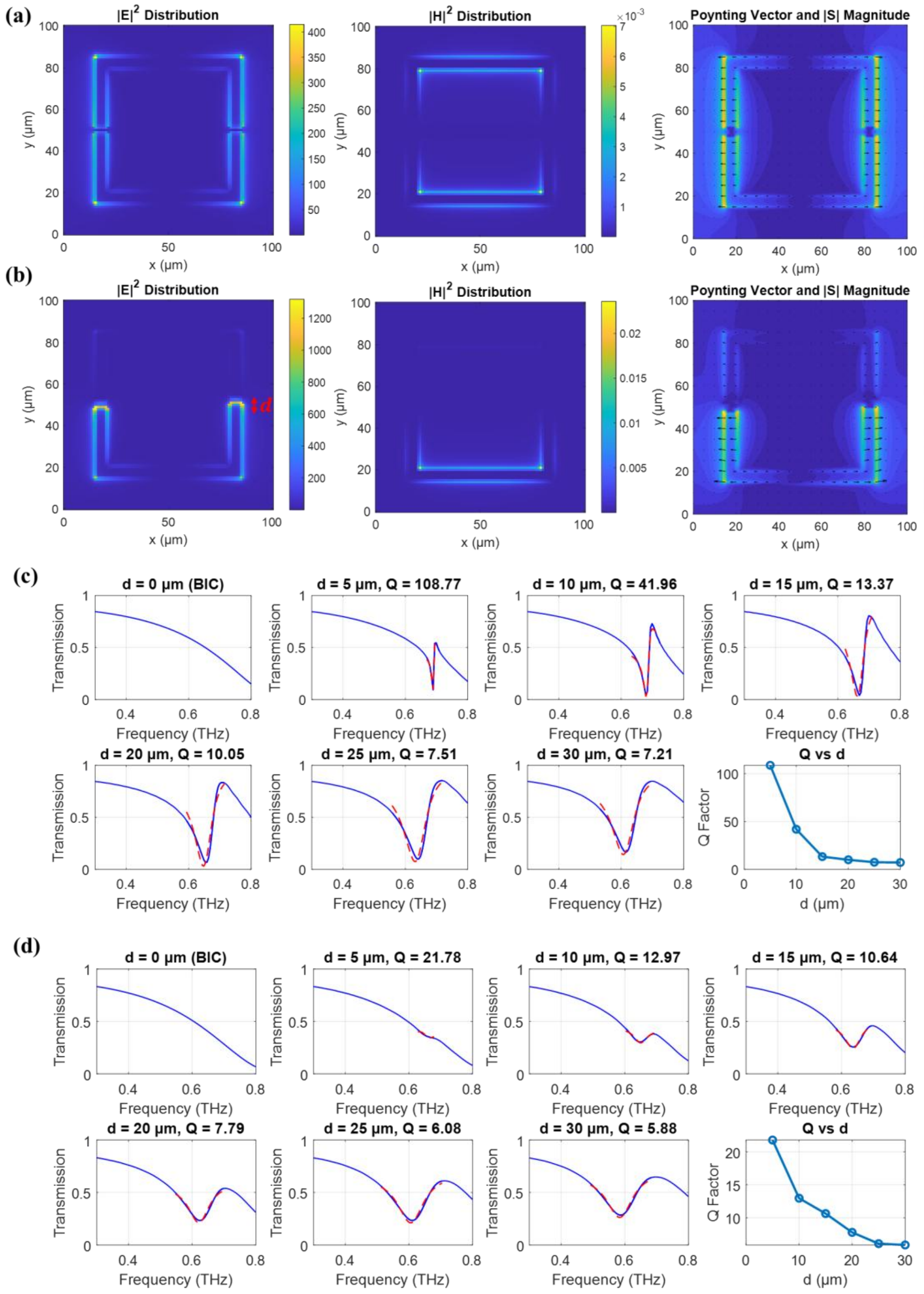

Figure S16. Physical analyses of BIC. (a) The near-field distributions and Poynting vector distribution when the metamaterial structure is symmetric ($d = 0$ μm). (b) The near-field distributions and Poynting vector distribution when the symmetry of the metamaterial structure is broken ($d = 2$ μm). (c) The Q factors with varying d when the metal material is set as PEC. (d) The Q factors with varying d when the metal material is set as Al.

3) Toroidal dipole

To analyze the near-field response of the classical metamaterial resonator Structure 6 [34] excited by a toroidal dipole, we perform simulations at different gap positions d and present the results in **Figure S17**. This figure includes the transmission spectra, electric field amplitude and phase distributions (component E_y), and the magnetic field distributions with overlaid vectors. When $d = 0$ μm (Figure S17(a)), a weak resonance is observed, with only slight field localization and no clear magnetic circulation. As d increases to 4 μm (Figure S17(b)), the resonance disappears, and the fields become more spread-out. This suggests that the internal current paths are not well formed, and the toroidal dipole cannot be excited. However, when d becomes larger, such as 8 μm and 12 μm in Figures S17(c) and S17(d), respectively, a strong resonance appears again. The electric field becomes tightly confined in certain regions, and the phase map shows a spiral-like rotation. At the same time, the magnetic field forms circular loops in the central area, indicating the generation of a toroidal magnetic moment. These patterns with strong localized electric fields around a circular magnetic structure are typical signs of toroidal dipole excitation. These results show that the resonance depends strongly on the shape and connection of the current paths inside the structure, which can be effectively tuned by changing the parameter d, consistent with the analysis in [34].

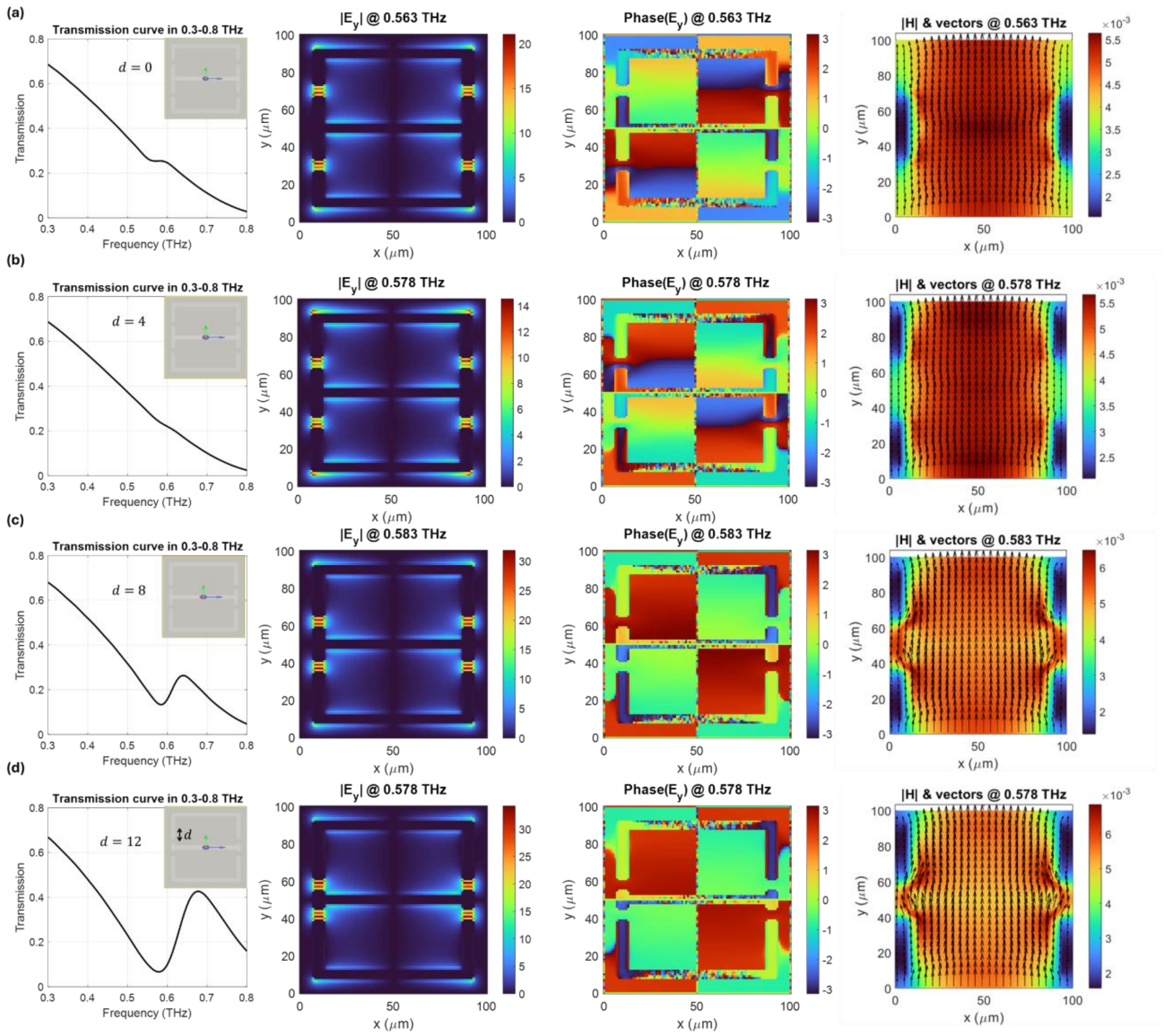

Figure S17. Electromagnetic field analysis of the classical resonator excited by toroidal dipole, including the transmission curves, amplitude and phase of the electric filed distribution E_y, and the magnetic field distribution H of the structure with the varying geometric parameter when (a) $d = 0$, (b) $d = 4$, (c) $d = 8$, and (d) $d = 12$.

4) EIT

To demonstrate the emergence of a classical bright–bright mode induced EIT effect in Structure 9 [31], we conduct simulations for the two sub-resonators and the combined resonator and analyse their near-field and transmission responses in **Figure S18**. As presented in Figure S18(a), sub-resonator 1 and sub-resonator 2 each exhibit distinct electric dipole resonances at different frequencies, as shown by their individual transmission dips and strong localized electric field distributions in Figure S18(b). When these two resonators are combined, their coupling leads to a hybrid mode response, as depicted in Figure S18(c). Specifically, the transmission spectrum, as shown in Figure S18(a), reveals a sharp transparency window (“A”)

situated between two pronounced dips ("B" and "C"), corresponding to the resonances of the individual bright modes. At this transparency frequency, the near-field distribution shows simultaneous excitation of both resonators, with their emitted fields interfering destructively in the far field. This interference suppresses radiation loss, enabling high transmission, which is a hallmark of the EIT phenomenon formed via the coherent coupling of two bright modes.

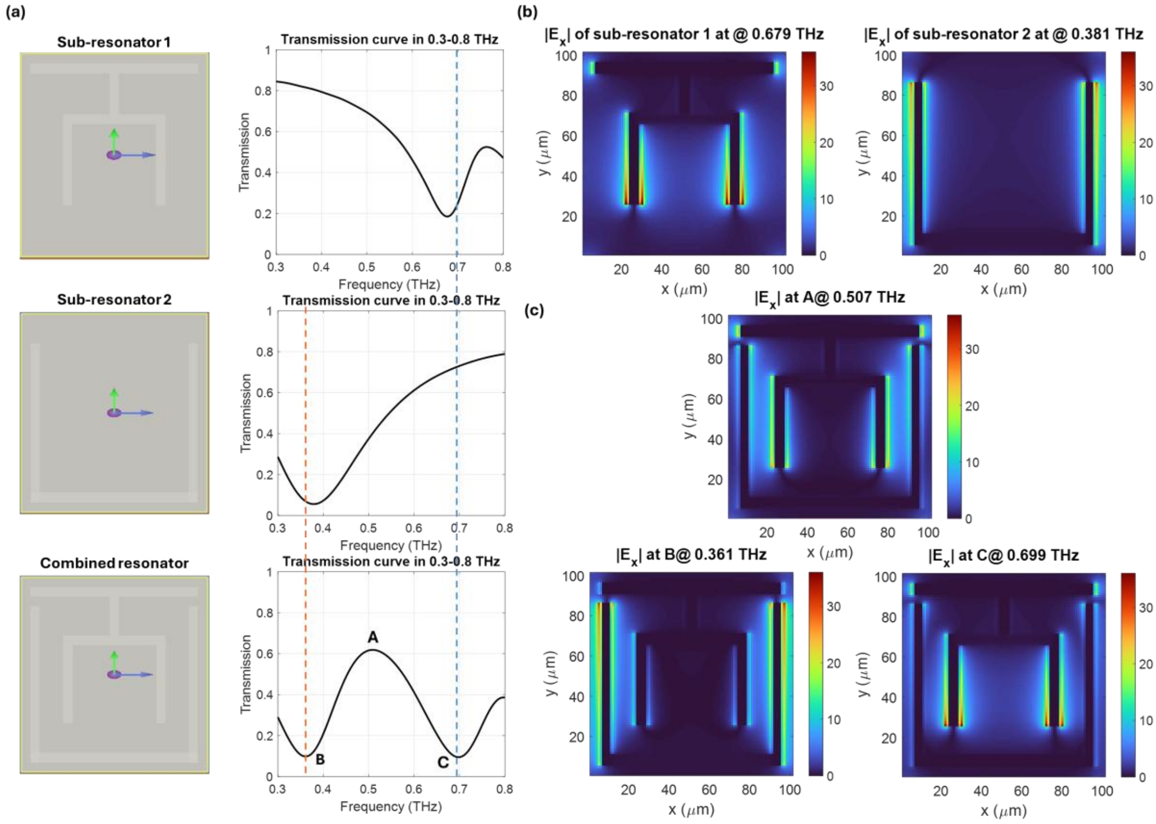

Figure S18. Near-field analysis of the two constituent resonators and their combined structure to verify the bright–bright mode induced EIT effect. (a) The transmission curves of the sub-resonators 1 and 2 as well as the combined resonator, where a pronounced transparency peak ("A") appears between the two dips ("B" and "C"). (b) The electric field distributions of the two sub-resonators at their respective resonance frequencies. (c) The electric field distribution of the combined structure at the peak ("A") and the two dips ("B" and "C").

Supplementary Note 13: Resonance Metric Comparison with Existing Works

To further compare with the FoMs of existing works, which is our goal of optimization in the proposed design active learning framework, we extract their FoM values from the transmission curves shown in [21, 22, 26, 29, 31, 33, 34] and listed them in **Table S4**. It should be noted that the FoM of the structure in [21] is intuitively provided. We can observe that the FoM realized by our proposed high-asymmetry structure is the highest. This demonstrates that our proposed design framework indeed outperforms the existing works on the resonance metrics and achieved high-FoM resonance.

Table S4. Comparative analysis of the resonance metrics FoM with existing works

Reference	Mechanism	Frequency (THz)	Asymmetry	Max. FoM
[21]	Fano	0.2-1.0	Low	6.2
[22]	Fano	0.45-0.55	Low	~3.0
[34]	Toroidal dipole	0.3-0.7	Low	~5.6
[31]	EIT	0.1-1.5	Low	~2.0
[33]	EIT	0.3-1.0	Low	~5.5
[26]	BIC	0.35-0.55	Low	~2.8
[29]	BIC	0.75-1.75	Low	~2.8
Ours	Data-driven	0.3-0.8	High	**7.7**

Supplementary Note 14: Difference between FoM and IFoM

The choice of FoM as the screening criterion in both the initial training dataset and the active-learning loop was based on practical and historical reasons. The effectiveness of FoM has been demonstrated in existing studies such as [21] as a standard metric to balance the resonance intensity and Q factor. On the other hand, the IFoM was designed as an evaluation metric. Unlike FoM, IFoM removes the effect of frequency dependence, allowing a fairer and more general comparison of resonance quality across different frequency bands. While FoM tends to reward sharper resonances but can favor low-frequency modes, IFoM corrects this bias by including frequency scaling. In short, FoM is used during the learning process to decide which candidates to keep, while IFoM is used afterward to judge and compare overall performance more objectively. The active-learning loop itself mainly relies on relative ranking, choosing samples from each batch that exceed a moving performance threshold.

We have conducted further experiments using IFoM as the selection criterion in the active learning process instead of FoM. To build the initial training dataset with high IFoM, we select 36 classical structures with IFoMs that are larger than 2.0 from the previous training dataset. For the active learning framework, we change the screening criterion in the second-step annotation and remain other parameters unchanged. For the training process, as the amount of initial training data samples becomes smaller than before, we generate 50 samples in each iteration and conduct 10 iterations of learning. The preliminary results are presented in **Figure S19**, showing that the active learning framework can also work when IFoM is used as the screening criterion. In Figure S19(a), we can see that the generated patterns become more diverse as the training continues. In each iteration, the training loss keeps going down, and the FID scores also decrease, which means the generative model effectively converges the training process. Figure S19(b) shows that the asymmetry of the generated structures is higher than that of the original dataset, and it first increases dramatically and then gently as the iteration increases. Figure S19(c) compares the FID scores using the fixed initial dataset as the reference. The values increase with more iterations, which suggests that the generated patterns are moving away from the initial set, and thus are exploring new design space. In Figure S19(d), the mean IFoM values of the generated structures also improve iteration by iteration and reach the highest at the fifth iteration. Figure S19(e) presents that both the average and maximum IFoM values of the generated structures are higher than those of the classical ones in the initial dataset. This means that our method can indeed produce designs with performance beyond the classical structures. Finally, Figure S19(f) provided examples of the top eight generated structures with

high IFoM values, along with their transmission spectra showing clear resonance dips. This demonstrates that the proposed framework can discover new structures with high-IFoM resonance even when the initial dataset is quite small.

In our study, we have tested three different screening criteria, the traditional FoM, the frequency-independent IFoM, and the Q factor at 0.5 THz (Note S11 and Figure S14). In all three cases, the framework consistently produced high-performance resonance structures and outperformed classical structures. This shows that the proposed approach does not rely on a single predefined metric but can be readily adapted to alternative criteria, opening the door for future extensions where new task-specific metrics may be introduced to guide the design process.

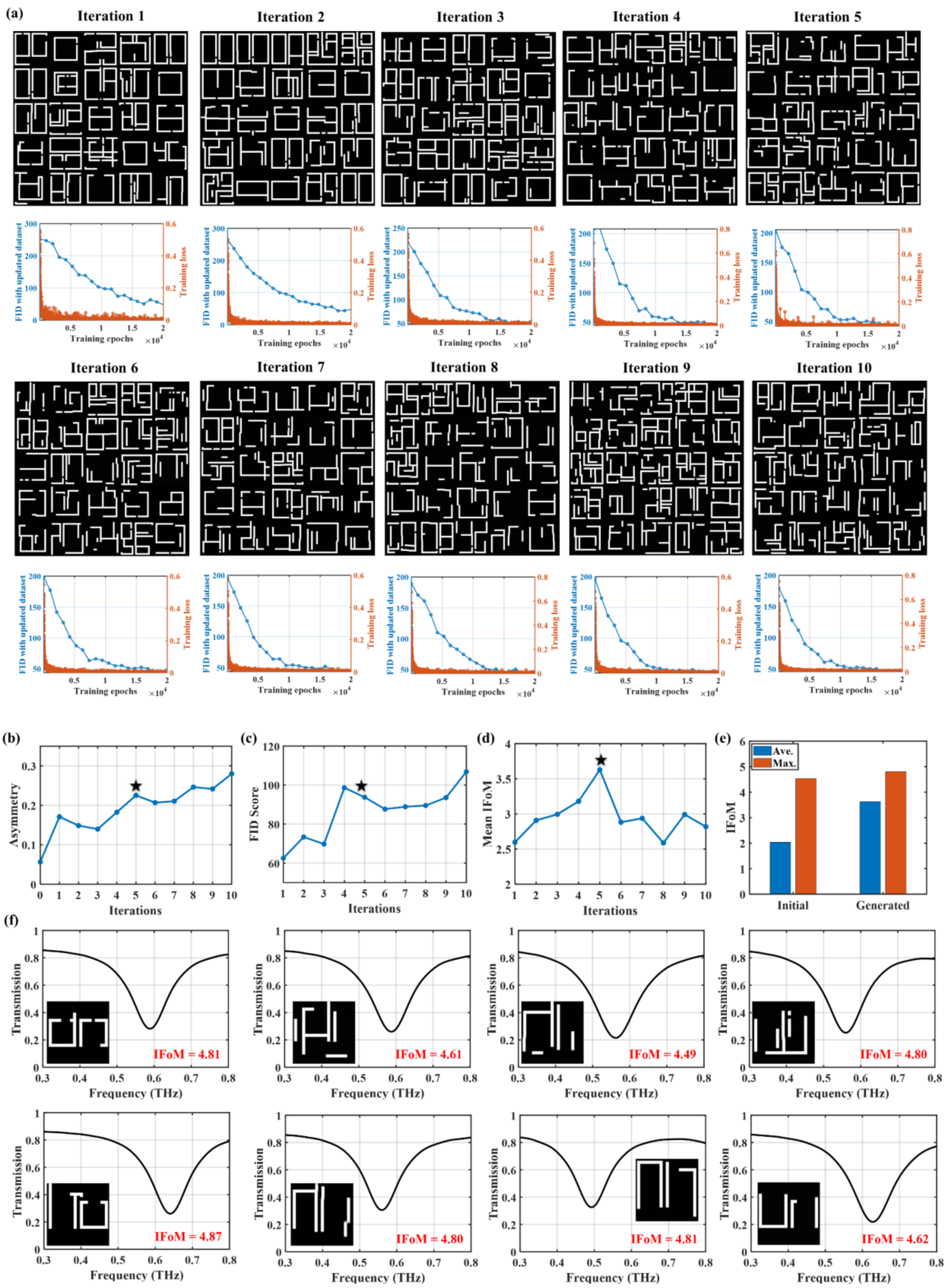

Figure S19. Preliminary results using IFoM as the screening criteria in the proposed active-learning screening. (a) The visualized patterns generated in each iteration and the training loss curve and the FID scores in each iteration. It should be noted that the FID scores here use the updated training dataset as reference. (b) The asymmetry of the initial dataset and the generated structures along with the iterations. (c)

The FID scores with iterations between the generated structures and the initial training dataset. It should be noted that the FID scores here use the fixed initial training dataset as reference. (d) The mean IFoM curves of the generated structures with the iterations. (e) Comparison of the mean IFoMs and maximum IFoMs of the generated structures and the classical structures adopted in the initial training dataset. (f) The generated structures with the maximum eight IFoMs and their transmission curves.

Supplementary References

[1] R. M. Woodward, B. E. Cole, V. P. Wallace, R. J. Pye, D. D. Arnone, E. H. Linfield, M. Pepper, *Phys. Med. Biol.* **2002**, *47*, 3853.

[2] E. Pickwell, V. P. Wallace, *J. Phys. D: Appl. Phys.* **2006**, *39*, R301.

[3] D. Suzuki, S. Oda, Y. Kawano, *Nat. Photonics* **2016**, *10*, 809.

[4] R. Singh, W. Cao, I. Al-Naib, L. Cong, W. Withayachumnankul, W. Zhang, *Appl. Phys. Lett.* **2014**, *105*, 171101.

[5] K. Shih, P. Pitchappa, M. Manjappa, C. P. Ho, R. Singh, C. Lee, *J. Appl. Phys.* **2017**, *121*, 023102.

[6] K. Shih, P. Pitchappa, L. Jin, C.-H. Chen, R. Singh, C. Lee, *Appl. Phys. Lett.* **2018**, *113*, 071105.

[7] S. Koenig, D. Lopez-Diaz, J. Antes, F. Boes, R. Henneberger, A. Leuther, A. Tessmann, R. Schmogrow, D. Hillerkuss, R. Palmer, T. Zwick, *Nat. Photonics* **2013**, *7*, 977.

[8] T. Nagatsuma, G. Ducournau, C. C. Renaud, *Nat. Photonics* **2016**, *10*, 371.

[9] J. F. Federici, B. Schulkin, F. Huang, D. Gary, R. Barat, F. Oliveira, D. Zimdars, *Semicond. Sci. Tech.* **2005**, *20*, S266.

[10] M. C. Kemp, P. F. Taday, B. E. Cole, J. A. Cluff, A. J. Fitzgerald, W. R. Tribe, in *Proc. Terahertz for Military and Security Applications*, Vol. 5070, SPIE, USA **2003**, 44.

[11] P. U. Jepsen, D. G. Cooke, M. Koch, *Laser Photon. Rev.* **2011**, *5*, 124.

[12] J. B. Baxter, G. W. Guglietta, *Anal. Chem.* **2011**, *83*, 4342.

[13] J. B. Pendry, A. J. Holden, D. J. Robbins, W. J. Stewart, *IEEE Trans. Microw. Theory Tech.* **1999**, *47*, 2075.

[14] T. J. Yen, W. J. Padilla, N. Fang, D. C. Vier, D. R. Smith, J. B. Pendry, D. N. Basov, X. Zhang, *Science* **2004**, *303*, 1494.

[15] H.-T. Chen, W. J. Padilla, J. M. Zide, A. C. Gossard, A. J. Taylor, R. D. Averitt, *Nature* **2006**, *444*, 597.

[16] C. Jansen, I. A. I. Al-Naib, N. Born, M. Koch, *Appl. Phys. Lett.* **2011**, *98*, 051109.

[17] Y. Lu, L. L. Hale, A. M. Zaman, S. J. Addamane, I. Brener, O. Mitrofanov, R. Degl'Innocenti, *ACS Photonics* **2023**, *10*, 2832.

[18] M. Zhu, Y.-S. Lin, C. Lee, *J. Appl. Phys.* **2014**, *116*, 173106.

[19] J. Niu, Y. Zhai, Q. Han, J. Liu, B. Yang, *Opt. Lett.* **2021**, *46*, 162.

[20] P. Liu, Z. Zhao, Y. Xue, X. Zhang, C. Jiang, R. T. Ako, H. Qin, S. Sriram, *Opt. Lett.* **2024**, *49*, 1301.

[21] L. Cong, M. Manjappa, N. Xu, I. Al-Naib, W. Zhang, R. Singh, *Adv. Opt. Mater.* **2015**, *3*, 1537.

[22] Y. K. Srivastava, M. Manjappa, L. Cong, W. Cao, I. Al-Naib, W. Zhang, R. Singh, *Adv. Opt. Mater.* **2016**, *4*, 457.

[23] L. Cong, R. Singh, *Adv. Opt. Mater.* **2019**, *7*, 1900383.

[24] T. C. W. Tan, E. Plum, R. Singh, *Adv. Opt. Mater.* **2020**, *8*, 1901572.

[25] Y. Wang, S. Jia, J. Qin, *Front. Phys.* **2021**, 8, 605125.

[26] X. Zhang, W. Shi, J. Gu, L. Cong, X. Chen, K. Wang, Q. Xu, J. Han, W. Zhang, *Opt. Express* **2022**, *30*, 29088.

[27] T. Zhang, X. Zhang, Y. Shen, Y. Xu, L. Luo, J. Gu, S. Yang, J. Xu, X. Deng, *APL Mater.* **2023**, *11*, 051102.

[28] Y. Hu, M. Tong, S. Hu, W. He, X. Cheng, T. Jiang, *Adv. Funct. Mater.* **2022**, *32*, 2203680.

[29] Z. Cui, Y. Wang, G. Sun, W. Chen, K. Zhang, X. Wang, *Opt. Lett.* **2023**, *48*, 4809.

[30] S. Yang, C. Tang, Z. Liu, B. Wang, C. Wang, J. Li, L. Wang, C. Gu, *Opt. Express* **2017**, *25*, 15938.

[31] B.-X. Wang, G. Duan, W. Lv, Y. Tao, H. Xiong, D.-Q. Zhang, G. Yang, F.-Z. Shu, *Nanoscale* **2023**, *15*, 18435.

[32] Y.-S. Lin, C.-Y. Huang, C. Lee, *IEEE J. Sel. Top. Quantum Electron.* **2015**, *21*, 2700207.

[33] P. Pitchappa, M. Manjappa, C. P. Ho, R. Singh, N. Singh, C. Lee, *Adv. Opt. Mater.* **2016**, *4*, 541.

[34] M. Gupta, V. Savinov, N. Xu, L. Cong, G. Dayal, S. Wang, W. Zhang, N. I. Zheludev, R. Singh, *Adv. Mater.* **2016**, *28*, 8206.

[35] X. Chen, W. Fan, X. Jiang, H. Yan, *J. Lightwave Technol.* **2021**, *40*, 2181.

[36] C. Xu, Z. Ren, J. Wei, C. Lee, *iScience* **2022**, *25*, 103799.

[37] W. J. Padilla, A. J. Taylor, C. Highstrete, M. Lee, R. D. Averitt, *Phys. Rev. Lett.* **2006**, *96*, 107401.

[38] J. Gu, R. Singh, X. Liu, X. Zhang, Y. Ma, S. Zhang, S. A. Maier, Z. Tian, A. K. Azad, H.-T. Chen, A. J. Taylor, J. Han, W. Zhang, *Nat. Commun.* **2012**, *3*, 1151.

[39] S. Savo, D. Shrekenhamer, W. J. Padilla, *Adv. Opt. Mater.* **2014**, *2*, 275.

[40] J. Wu, Z. Shen, S. Ge, B. Chen, Z. Shen, T. Wang, C. Zhang, W. Hu, K. Fan, W. Padilla, Y. Lu, B. Jin, J. Chen, P. Wu, *Appl. Phys. Lett.* **2020**, *116*, 131104.

[41] P. Pitchappa, A. Kumar, S. Prakash, H. Jani, T. Venkatesan, R. Singh, *Adv. Mater.* **2019**, *31*, 1808157.

[42] P. Pitchappa, A. Kumar, S. Prakash, H. Jani, R. Medwal, M. Mishra, R. S. Rawat, T. Venkatesan, N. Wang, R. Singh, *Adv. Funct. Mater.* **2021**, *31*, 2100200.

[43] L. Ju, B. Geng, J. Horng, C. Girit, M. Martin, Z. Hao, H. A. Bechtel, X. Liang, A. Zettl, Y. R. Shen, F. Wang, *Nat. Nanotechnol.* **2011**, *6*, 630.

[44] S. H. Lee, M. Choi, T.-T. Kim, S. Lee, M. Liu, X. Yin, H. K. Choi, S. S. Lee, C.-G. Choi, S.-Y. Choi, X. Zhang, B. Min, *Nat. Mater.* **2012**, *11*, 936.

[45] P. Pitchappa, A. Kumar, R. Singh, C. Lee, N. Wang, *J. Micromech. Microeng.* **2021**, *31*, 113001.

[46] Y.-S. Lin, C. Lee, *Appl. Phys. Lett.* **2014**, *104*, 251914.

[47] F. Ma, Y.-S. Lin, X. Zhang, C. Lee, *Light Sci. Appl.* **2014**, *3*, 1.

[48] P. Pitchappa, C. P. Ho, Y.-S. Lin, P. Kropelnicki, C.-Y. Huang, N. Singh, C. Lee, *Appl. Phys. Lett.* **2014**, *104*, 151104.

[49] C. P. Ho, P. Pitchappa, Y.-S. Lin, C.-Y. Huang, P. Kropelnicki, C. Lee, *Appl. Phys. Lett.* **2014**, *104*, 161104.

[50] P. Pitchappa, C. P. Ho, L. Dhakar, C. Lee, *Optica* **2015**, *2*, 571.

[51] P. Pitchappa, M. Manjappa, C. P. Ho, R. Singh, N. Singh, C. Lee, *Appl. Phys. Lett.* **2016**, *109*, 211103.

[52] P. Pitchappa, C. P. Ho, L. Cong, R. Singh, N. Singh, C. Lee, *Adv. Opt. Mater.* **2016**, *4*, 391.

[53] L. Cong, P. Pitchappa, C. Lee, R. Singh, *Adv. Mat.* **2017**, *29*, 1700733.

[54] L. Cong, P. Pitchappa, Y. Wu, L. Ke, C. Lee, N. Singh, H. Yang, R. Singh, *Adv. Opt. Mater.* **2017**, *5*, 1600716.

[55] M. Manjappa, P Pitchappa, N. Wang, C. Lee, R. Singh, *Adv. Opt. Mater.* **2018**, *6*, 1800141.

[56] M. Manjappa, P. Pitchappa, N. Singh, N. Wang, N. Zheludev, C. Lee, R. Singh, *Nat. Commun.* **2018**, *9*, 4056.

[57] P. Pitchappa, M. Manjappa, H. N. S. Krishnamoorthy, Y. Chang, C. Lee, R. Singh, *Appl. Phys. Lett.* **2017**, *111*, 261101.

[58] P. Pitchappa, A. Kumar, H. Liang, S. Prakash, N. Wang, A. A Bettiol, T. Venkatesan, C. Lee, R. Singh, *Adv. Optical Mater.* **2020**, *8*, 2000101.

[59] Z. Liu, D. Zhu, L. Raju, W. Cai, *Adv. Sci.* **2021**, *8*, 2002923.

[60] M. K. Chen, X.Liu, Y. Sun, D. P. Tsai, *Chem. Rev.* **2022**, *122*, 15356.

[61] K. Vijay Mishra, A. M. Elbir, A. I. Zaghloul, *Advances in Electromagnetics Empowered by Artificial Intelligence and Deep Learning*, Wiley-IEEE Press, **2023**, 281−317.

[62] C. Qian, I. Kaminer, H. Chen, *Nat. Commun.* **2025**, *16*, 1154.

[63] W. Ma, F. Cheng, Y. Liu, *ACS Nano* **2018**, *12*, 6326.

[64] I. Malkiel, M. Mrejen, A. Nagler, U. Arieli, L. Wolf, H. Suchowski, *Light Sci. Appl.* **2018**, *7*, 60.

[65] Z. Hou, T. Tang, J. Shen, C. Li, F. Li, *Nanoscale Res. Lett.* **2020**, *15*, 83.

[66] J. H. Han, Y.-C. Lim, R. M. Kim, J. Lv, N. H. Cho, H. Kim, S. D. Namgung, S. W. Im, K. T. Nam, *ACS Nano* **2023**, *17*, 2306.

[67] H.-T. Lee, J. Kim, J. S. Lee, M. Yoon, H.-R. Park, *Nano Lett.* **2023**, *23*, 11685.

[68] P. Peng, Z. Liu, C. Dai, X. He, D. Wang, Z. Dang, Y. Chen, Y. Dai, H. Zhang, S. Sun, L. Zhou, Z. Fang, *Adv. Optical Mater.* **2024**, *12*, 2300158.

[69] Q. Zhang, C. Liu, X. Wan, L. Zhang, S. Liu, Y. Yang, T. J. Cui, *Adv. Theory Simul.* **2019**, *2*, 1800132.

[70] R. Zhu, T. Qiu, J. Wang, S. Sui, C. Hao, T. Liu, Y. Li, M. Feng, A. Zhang, C.-W. Qiu, S. Qu, *Nat. Commun.* **2021**, *12*, 2974.

[71] Y. Jia, C. Qian, Z. Fan, T. Cai, E.-P. Li, H. Chen, *Light Sci. Appl.* **2023**, *12*, 82.

[72] Z. Liu, D. Zhu, S. P. Rodrigues, K.-T. Lee, W. Cai, *Nano Lett.* **2018**, *18*, 6570.

[73] S. So, J. Rho, *Nanophotonics* **2019**, *8*, 1255.

[74] S. An, B. Zheng, H. Tang, M. Y. Shalaginov, L. Zhou, H. Li, M. Kang, K. A. Richardson, T. Gu, J. Hu, C. Fowler, H. Zhang, *Adv. Optical Mater.* **2021**, *9*, 2001433.

[75] J. Wang, S. Chen, Y. Qiu, X. Chen, J. Shen, C. Li, *Micromachines* **2023**, *14*, 1925.

[76] W. Ma, F. Cheng, Y. Xu, Q. Wen, Y. Liu, *Adv. Mater.* **2019**, *31*, 1901111.

[77] I. Tanriover, D. Lee, W. Chen, K. Aydin, *ACS Photonics* **2022**, *10*, 875.

[78] N. Zhang, F. Gao, R. Wang, Z. Shen, D. Han, Y. Cui, L. Zhang, C. Chang, C.-W. Qiu, X. Chen, *Adv. Mater.* **2025**, *37*, 2411490.

[79] J. Ho, A. Jain, P. Abbeel, *Proc. NeurIPS* **2020**, *33*, 6840.

[80] J. Sohl-Dickstein, E. Weiss, N. Maheswaranathan, S. Ganguli, *Proc. ICML* **2015**, *37*, 2256.

[81] J. R. Hershey, P. A. Olsen, *Proc. Int. Conf. Acoust.* **2007**, *4*, IV-317.

[82] C. Luo, *(Preprint) arXiv*, arXiv:2208.11970, submitted: Aug. **2022**.

[83] S. J. Park, R. S. Parker-Jervis, J. E. Cunningham, *Adv. Theory Simul.* **2022**, *5*, 2100428.

[84] D. P. Kingma, M. Welling, in *Proc. Int. Conf. Learn. Represent.* (ICLR), **2014**, arXiv:1312.6114.

[85] Y. Pu, Z. Gan, R. Henao, X. Yuan, C. Li, A. Stevens, L. Carin, in *Adv. Neural Inf. Process. Syst.* (NeurIPS), Vol. 29, **2016**.

[86] I. Goodfellow, J. Pouget-Abadie, M. Mirza, B. Xu, D. Warde-Farley, S. Ozair, A. Courville, Y. Bengio, in *Adv. Neural Inf. Process. Syst.* (NeurIPS), Vol. 27, **2014**.

[87] A. Radford, L. Metz, S. Chintala, in *Proc. Int. Conf. Learn. Represent.* (ICLR), **2016**, arXiv:1511.06434.

[88] R. Zhang, P. Isola, A. A. Efros, E. Shechtman, O. Wang, in *Proc. IEEE/CVF Conf. Comput. Vis. Pattern Recognit.* (CVPR), Salt Lake City, UT, USA, **2018**, pp. 586–595.
